# Long-term safety and efficacy of lentiviral hematopoietic stem/progenitor cell gene therapy for Wiskott–Aldrich syndrome

**DOI:** 10.1038/s41591-021-01641-x

**Published:** 2022-01-24

**Authors:** A. Magnani, M. Semeraro, F. Adam, C. Booth, L. Dupré, E. C. Morris, A. Gabrion, C. Roudaut, D. Borgel, A. Toubert, E. Clave, C. Abdo, G. Gorochov, R. Petermann, M. Guiot, M. Miyara, D. Moshous, E. Magrin, A. Denis, F. Suarez, C. Lagresle, A. M. Roche, J. Everett, A. Trinquand, M. Guisset, J. Xu Bayford, S. Hacein-Bey-Abina, A. Kauskot, R. Elfeky, C. Rivat, S. Abbas, H. B. Gaspar, E. Macintyre, C. Picard, F. D. Bushman, A. Galy, A. Fischer, E. Six, A. J. Thrasher, M. Cavazzana

**Affiliations:** 1 Department of Biotherapy, Hôpital Universitaire Necker-Enfants Malades, Groupe Hospitalier Paris Centre, Assistance Publique-Hôpitaux de Paris, Paris, France; 2 Biotherapy Clinical Investigation Center, Groupe Hospitalier Universitaire Paris Centre, Assistance Publique-Hôpitaux de Paris, INSERM CIC 1416, Paris, France; 3 Clinical Investigation Center CIC 1419, Hôpital Universitaire Necker-Enfants Malades, Groupe Hospitalier Paris Centre, Université de Paris, Assistance Publique-Hôpitaux de Paris, Paris, France; 4 INSERM, UMR_S1176, Université Paris-Saclay, Le Kremlin-Bicêtre, France; 5 Department of Paediatric Immunology, Great Ormond Street Hospital, London, UK; 6 Molecular and Cellular Immunology Section, UCL Great Ormond Street Institute of Child Health, London, UK; 7 Toulouse Institute for Infectious and Inflammatory Diseases (INFINITy), INSERM, CNRS, Toulouse III Paul Sabatier University, Toulouse, France; 8 Ludwig Boltzmann Institute for Rare and Undiagnosed Diseases, Vienna, Austria; 9 Department of Dermatology, Medical University of Vienna, Vienna, Austria; 10 Institute of Immunity and Transplantation, University College London, London, UK; 11 Department of Immunology, Royal Free London Hospitals NHS Foundation Trust, London, UK; 12 Laboratoire d’Hématologie, Assistance Publique-Hôpitaux de Paris, Hôpital Necker-Enfants Malades, Paris, France; 13 EMiLy, INSERM U1160, Institut de Recherche Saint Louis, Université de Paris, Paris, France; 14 Laboratoire d’Immunologie et d’Histocompatibilité, Hôpital Saint-Louis, Assistance Publique-Hôpitaux de Paris, Paris, France; 15 Institut Necker-Enfants Malades (INEM), INSERM U1151, Université Paris Descartes Sorbonne Cité, Paris, France; 16 Laboratory of Onco-Hematology, Assistance Publique-Hôpitaux de Paris, Necker-Enfants Malades University Hospital, Paris, France; 17 Département d’Immunologie, Groupement Hospitalier Pitié-Salpêtrière, Assistance Publique-Hôpitaux de Paris, Paris, France; 18 Centre d’Immunologie et des Maladies Infectieuses-Paris (CIMI-Paris), INSERM, Sorbonne Université, Paris, France; 19 Platelet Immunology Department, INTS, Paris, France; 20 Department of Pediatric Immunology, Hematology and Rheumatology, Assistance Publique-Hôpitaux de Paris, Necker-Enfants Malades University Hospital, Paris, France; 21 Imagine Institute, Université Paris Centre, Paris, France; 22 Human Lymphohematopoiesis Laboratory, Imagine Institute, INSERM UMR 1163, Université de Paris, Paris, France; 23 Service d’Hématologie Adultes, Hôpital Necker-Enfants Malades, Assistance Publique-Hôpitaux de Paris, Centre Université de Paris, Paris, France; 24 Department of Microbiology, University of Pennsylvania School of Medicine, Philadelphia, PA USA; 25 Unité des Technologies Chimiques et Biologiques pour la Santé, CNRS, INSERM, Université de Paris, Paris, France; 26 Clinical Immunology Laboratory, Groupe Hospitalier Universitaire Paris-Sud, Hôpital Kremlin-Bicêtre, Assistance Publique-Hôpitaux de Paris, Le Kremlin-Bicêtre, France; 27 Généthon, Evry, France; 28 Study Center for Primary Immunodeficiencies, Assistance Publique-Hôpitaux de Paris, Necker-Enfants Malades University Hospital, Paris, France; 29 Integrare Research Unit UMRS951, Université Paris-Saclay, Université d’Evry, Inserm, Genethon, Evry, France; 30 Collège de France, Paris, France

**Keywords:** Targeted gene repair, Primary immunodeficiency disorders

## Abstract

Patients with Wiskott–Aldrich syndrome (WAS) lacking a human leukocyte antigen-matched donor may benefit from gene therapy through the provision of gene-corrected, autologous hematopoietic stem/progenitor cells. Here, we present comprehensive, long-term follow-up results (median follow-up, 7.6 years) (phase I/II trial no. NCT02333760) for eight patients with WAS having undergone phase I/II lentiviral vector-based gene therapy trials (nos. NCT01347346 and NCT01347242), with a focus on thrombocytopenia and autoimmunity. Primary outcomes of the long-term study were to establish clinical and biological safety, efficacy and tolerability by evaluating the incidence and type of serious adverse events and clinical status and biological parameters including lentiviral genomic integration sites in different cell subpopulations from 3 years to 15 years after gene therapy. Secondary outcomes included monitoring the need for additional treatment and T cell repertoire diversity. An interim analysis shows that the study meets the primary outcome criteria tested given that the gene-corrected cells engrafted stably, and no serious treatment-associated adverse events occurred. Overall, severe infections and eczema resolved. Autoimmune disorders and bleeding episodes were significantly less frequent, despite only partial correction of the platelet compartment. The results suggest that lentiviral gene therapy provides sustained clinical benefits for patients with WAS.

## Abstract (web summary)

Long-term monitoring of patients with Wiskott–Aldrich syndrome following lentiviral gene therapy shows a safe profile and a reduction in the frequency of autoimmune manifestations and bleeding events, despite incomplete platelet reconstitution.

## Main

Wiskott–Aldrich syndrome (WAS) is a complex X-linked disorder caused by loss-of-function mutations in the *WAS* gene encoding WAS protein (WASp), a key regulator of the actin cytoskeleton in hematopoietic cells^1^. WASp deficiency causes characteristic microthrombocytopenia and lymphoid–myeloid dysfunction, the severity of which usually depends on the residual levels of WASp expression and function. WAS affects all the hematopoietic cellular compartments, which explains the broad range of associated clinical manifestations^1–3^. The Zhu score^4^ is commonly used to stratify disease severity. The most severe phenotype is characterized by susceptibility to severe infection, bleeding, eczema, autoimmunity and a risk of malignancy^5,6^. Without a curative treatment for WAS, most patients lacking functional WASp do not survive beyond their second or third decade of life^7^.

The most severe, early-onset, life-threatening form of WAS is characterized by profound, treatment-refractory thrombocytopenia^6^. At the other end of the WAS severity spectrum, X-linked thrombocytopenia (XLT) represents a milder disease entity. It is primarily characterized by microthrombocytopenia, although patients with XLT can also develop classical complications of WAS.

The first-line treatment for WAS is allogeneic hematopoietic stem cell transplantation (HSCT) with a human leukocyte antigen (HLA)-compatible donor. Although transplantation was initially associated with substantial mortality and morbidity rates, outcomes have substantially improved^8–11^. In the most recently described cohort of patients with WAS having undergone HSCT^12^, the overall survival rate was 94% for those treated before the age of 5 years. The rate fell to 66% in those treated at an older age. The outcome after HSCT is influenced by the level of donor engraftment. Mixed donor chimerism after HSCT is associated with an increased risk of poor immune reconstitution and autoimmunity. In particular, donor myeloid engraftment below 50% is associated with persistent thrombocytopenia^8,9,11,12^. In addition, between 10% and 20% of long-term survivors have active autoimmune complications, which are closely linked to the level of mixed chimerism (<70%)^8,9^.

For patients lacking a suitable donor for HSCT, other treatment options have been developed. These include haplo-identical HSCT and gene therapy via the infusion of gene-corrected autologous hematopoietic stem cells (HSCs). Haplo-identical transplantation is an attractive option because a family member is almost always available as a donor for pediatric patients. Moreover, the high morbidity and mortality rates initially associated with this approach^8^ are now falling^11–13^.

The gene therapy approach bypasses many of the remaining problems associated with haplo-identical HSCT, in particular the toxicity of a full conditioning regimen and the risk of graft-versus-host disease. A rationale for gene therapy has been provided by natural somatic reversion events reported in a number of patients with WAS^14–16^. In such cases, the accumulation of WASp-expressing lymphocytes constitutes direct evidence of the in vivo selective advantage of genetically competent cells. A previous trial using a gamma retroviral vector with a viral promoter provided evidence for the efficacy of gene therapy for WAS^17^. Unfortunately, this treatment was associated with serious adverse events related to vector-mediated insertional mutagenesis in all of the patients^18^. To minimize mutagenic side effects, subsequent phase I/II clinical trials have been based on the use of a self-inactivating lentiviral vector expressing WASp under the control of a minimal endogenous promoter^19–21^. These studies have confirmed the efficacy of gene therapy for WAS, and no major safety concerns were highlighted.

We have previously reported the results of 9- to 42-month follow-up of seven pediatric patients with severe WAS enrolled in non-randomized, open-label, phase I/II gene therapy clinical studies (nos. NCT01347346 and NCT01347242) (ref. ^20^). Here, we report comprehensive, long-term follow-up efficacy and safety data (4–9 years after gene therapy; median, 7.6 years) for these and two additional patients (including a young adult patient) enrolled after the first report.

## Results

### Clinical presentation

We previously reported on non-randomized, open-label, phase I/II clinical studies (based on a lentiviral gene therapy vector) involving seven pediatric patients (referred to hereafter as patients 1–7) with severe WAS. The patients were treated at Necker-Enfants Malades Hospital (Paris, France) and at Great Ormond Street Hospital (London, UK)^20^. One patient (patient 3) died 7 months after gene therapy from pre-existing complications of infection, as reported previously^20^. Since the initial report, two additional patients treated at the Royal Free London Hospital and Great Ormond Street Hospital have been followed for at least 4 years after gene therapy and were included in the present study (patients 8 and 9). Patient 8 presented with severe cutaneous vasculitis, arthropathy, lymphoproliferation and chronic kidney disease requiring intensive immunosuppressive treatment and splenectomy before gene therapy^22^. Patient 9 had presented with gastrointestinal bleeding, severe eczema, recurrent infections, cytomegalovirus viremia and failure to thrive. All but one of the patients (patient 6) had a WAS score of 5. The patients’ clinical characteristics are summarized in Supplementary Table 1.

### Hematopoietic stem/progenitor cell engineering and transplantation

Autologous hematopoietic stem/progenitor cells (HSPCs) were transduced ex vivo with the LV-w1.6 WASp self-inactivating lentiviral vector^23^ and immediately reinfused without cryopreservation, as described previously^20^. The graft was collected from mobilized peripheral blood (*n* = 4) or bone marrow (*n* = 4) (Supplementary Table 1). At the time of gene therapy, the median patient age was 5.25 years (range, 0.8–30 years). The median vector copy number (VCN) per CD34^+^ cell in the drug product was 0.91 (range, 0.6–2.8). All of the patients underwent non-myeloablative conditioning with busulfan and fludarabine, as described previously^20^, together with anti-CD20 therapy in one patient (patient 7). The median dose of CD34^+^ cells infused was 7.05 × 10^6^ (range, 2–15 × 10^6^) per kilogram of body weight. No serious adverse events were recorded during or after the cell infusion. Neither delayed neutrophil recovery nor a requirement for granulocyte colony-stimulating factor was observed after transplantation.

### Clinical outcomes after gene therapy

**Extended Data Fig. 1:**
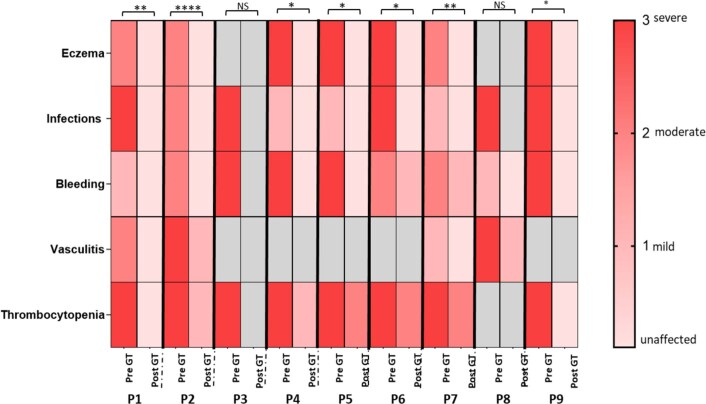
Clinical and laboratory features of patients with WAS before and after GT. Clinical and laboratory features of patients with WAS before and after GT: light pink: no disease; pink: mild disease; light red: moderate disease; red: severe disease. Eczema was graded with the SCORAD tool24. Infections and bleeding were graded in terms of recurrence and severity. Vasculitis was assessed according to the WAS severity scoring system4. Thrombocytopenia was defined as follows: grade 3 for a platelet count <10,000/µL; grade 2 between 10,000/µL and 20,000/µL; grade 1 between 20,000/µL and 50,000/µL. Grey box: not applicable. Statistical significance was determined by t test (***p < 0.001); NS: not significant.

After gene therapy, the patients were regularly evaluated over a period of at least 4 years. At the time of this interim analysis (cut-off, 1 October 2020), the median follow-up was 7.6 years (range, 4–9 years). The patients’ clinical outcomes are summarized in Supplementary Table 1 and Extended Data Fig. 1.

The progressive reconstitution of hematopoietic lineages in all of the patients was accompanied by a reduction in the frequency and severity of infections. Patient 7 had fluctuating reactivation of Epstein–Barr virus infection, but this was already present before gene therapy and did not require treatment. All of the patients discontinued their pre-gene therapy anti-infection prophylaxis, except for penicillin after splenectomy (and following myeloablative chemotherapy for UK patients). Five patients (patients 4, 5, 6, 8 and 9) discontinued immunoglobulin replacement therapy between 12 and 24 months after gene therapy. Eczema manifestations resolved in all but one patient (patient 7), who nevertheless had a marked reduction in the severity of eczema, with a decrease in the SCORing Atopic Dermatitis score^24^ from 52 to 15.

Prior to gene therapy, all of the patients had severe microthrombocytopenia, in some cases associated with life-threatening episodes of cerebral or gastrointestinal bleeding (patients 2, 4, 5, 7 and 9). After gene therapy, none of the patients had spontaneous, severe bleeding despite below-normal platelet counts and none required platelet transfusions.

Patient 8 was treated at the age of 30 years. After gene therapy, autoimmune manifestations, inflammatory manifestations and infections were markedly less frequent. He was able to discontinue immunoglobulin replacement therapy and immunosuppressants and showed protective levels of post-vaccination antibody titers. Immune reconstitution and gene marking levels were stable. Four years after gene therapy, this splenectomized patient died of concomitant pneumococcal sepsis and H1N1 influenza.

Patient 9 was treated at the age of 11 months. Twenty-one months after gene therapy, he presented with hematuria, proteinuria and edema. He was diagnosed with nephrotic syndrome, and a renal biopsy showed mild diffuse mesangial proliferative glomerulonephritis, immune complex-mediated. He was successfully treated with steroids, rituximab and mycophenolate mofetil. He is no longer taking immunosuppressants and is in good health.

With regard to inflammatory manifestations, patient 2 had developed very debilitating lower extremity vasculitis before gene therapy, which required wheelchair use. He underwent gene therapy at 16 years of age, followed by a marked improvement in vasculitis. He recovered the ability to walk but still had sequelae, including relapsing–remitting episodes of pain that partially responded to non-steroidal anti-inflammatory drugs and immunoglobulin replacement therapy. A marked improvement was observed after treatment with the human interleukin-1 receptor antagonist anakinra (100 mg per day); this treatment is ongoing and well tolerated.

Patient 4 had a single episode of single-joint (knee) inflammation 5 years after gene therapy, but recovered fully after conventional treatment with anti-inflammatory drugs. Three years later, he presented with moderate swelling of the ankles after an influenza vaccination, but this fully resolved after a short steroid treatment.

### Myeloid reconstitution after gene therapy

**Extended Data Fig. 2:**
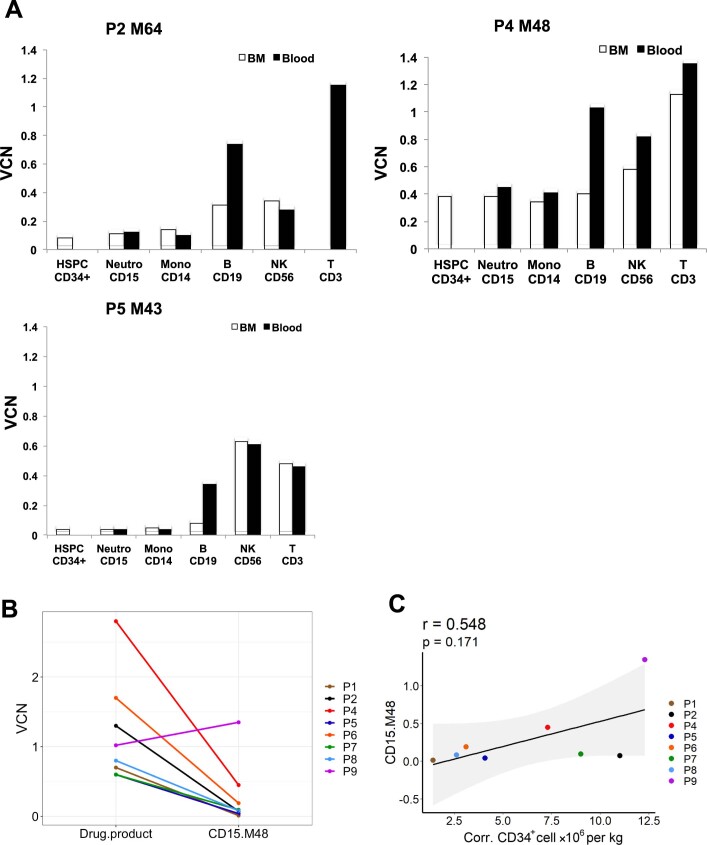
Gene marking level. Panel A, the VCN in the bone marrow (BM, white) subpopulations versus the peripheral blood (black) subpopulations in patients P2, P4 and P5; Panel B, VCN levels in the initial CD34 + drug product versus myeloid CD15 + cells 48 months after GT. Panel C, The Spearman correlation between the level of gene marking in CD15 + 48 months after GT and the number of corrected CD34 + cells infused per kilogram, for each patient. The pvalue (p) was calculated by using two-sided Spearman’s rank correlation test, r is Spearman’s rank correlation coefficient. A regression line is represented in black, the confidence interval in grey.

All of the patients had stable engraftment in the first 3 weeks after gene therapy. The level of gene marking in CD15^+^ and CD14^+^ myeloid cells reached a median of 8.9% (range, 1–100%) after 4 years of follow-up and corresponded to the level of gene marking in the engrafted CD34^+^ HSPCs in the bone marrow (Extended Data Fig. 2a). Except in patient 9, this level did not increase further over time and was markedly lower than in the drug product (Extended Data Fig. 2b). The level of gene marking in CD15^+^ cells tended to correlate with the number of corrected CD34^+^ cells per kilogram infused (Extended Data Fig. 2c).

### T cell immune reconstitution after gene therapy

**Fig. 1:**
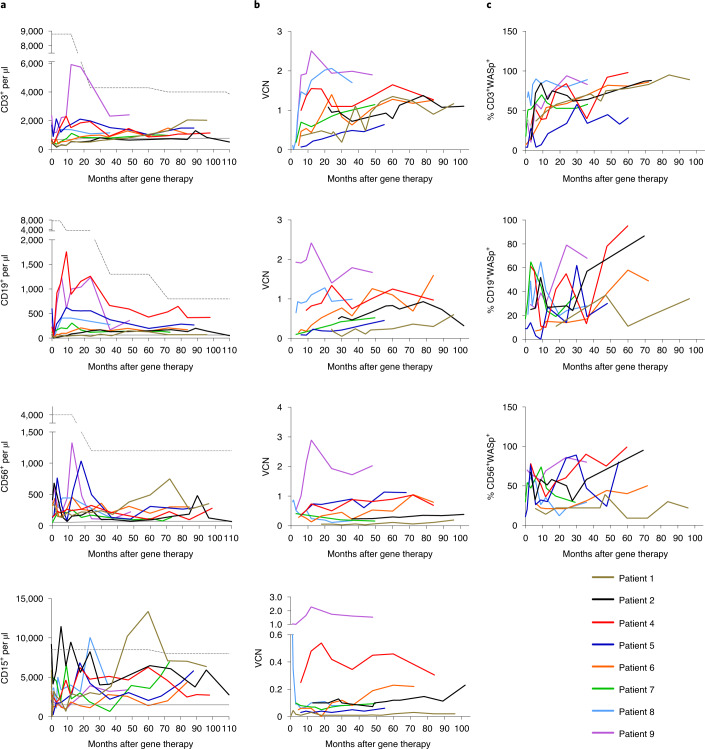
Immune reconstitution after gene therapy. **a**, Measurement of the total CD3^+^ T cell, CD19^+^ B cell, CD56^+^ natural killer (NK) cell and CD15^+^ neutrophil counts in blood at regular time points after gene therapy. Age-matched reference values are indicated by the gray lines (solid line, lower value; dashed line, upper value). **b**, Measurement of the VCN per cell at regular time points after gene therapy as a guide to the level of gene marking in CD3^+^ T cells, CD19^+^ B cells, CD56^+^ NK cells and CD15^+^ neutrophils (top to bottom). **c**, Change over time in the proportion of WASp-positive CD3^+^ T cells, CD19^+^ B cells and CD56^+^ NK cells after gene therapy.

**Fig. 2:**
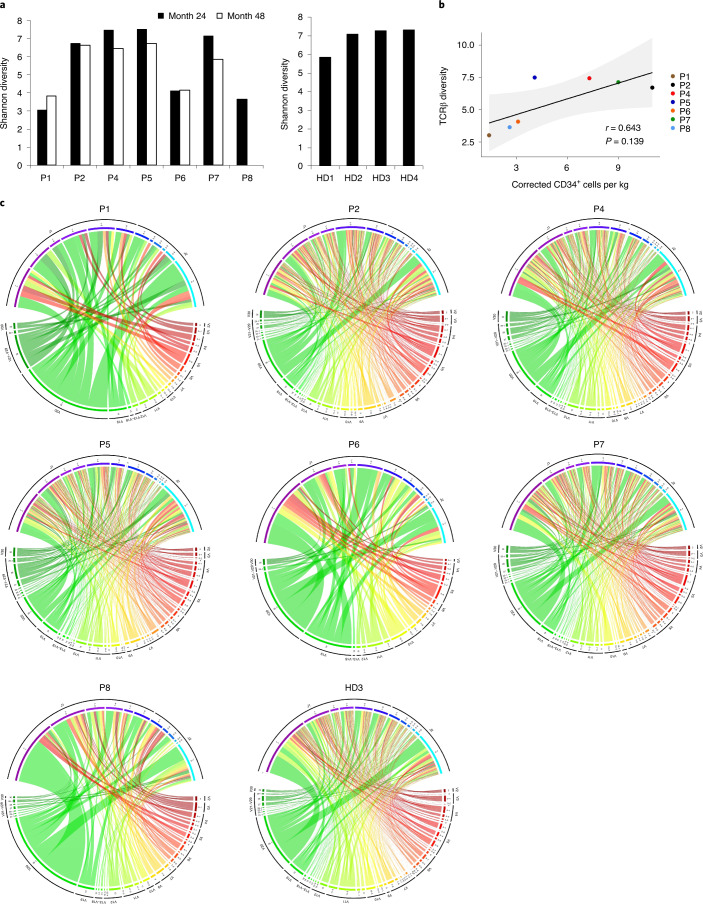
TCRβ repertoire analysis. **a**, Shannon diversity of the TCRβ clonotypes analyzed in patients with WAS (P1, P2, P4–P8) at months 24 and 48 of post-gene therapy follow-up (left) and in four healthy donors (HD1–HD4) (right). **b**, The Spearman correlation between the TCRβ Shannon diversity at month 24 and the number of corrected CD34^+^ cells infused per kilogram, for each patient. The *P* value was calculated using the two-sided Spearman’s rank correlation test, and *r* is Spearman’s rank correlation coefficient. A regression line is represented in black and the 95% confidence interval is shown in gray. **c**, Circos plots showing V-gene and J-gene combinations in the repertoires analyzed in patients with WAS at month 48 of post-gene therapy follow-up (except for P8, shown at month 24) and in one healthy donor (HD3). The colored arcs define distinct Vβ and Jβ genes. The ribbon thickness is proportional to the frequency of a given combination.

**Extended Data Fig. 3:**
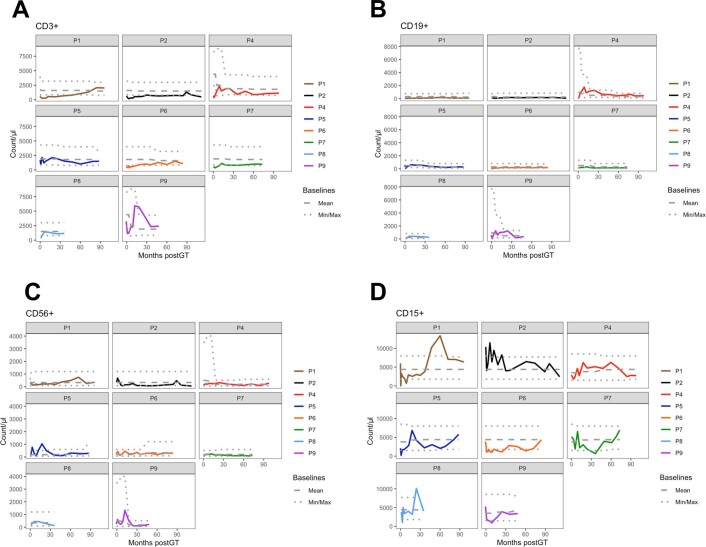
Immunological reconstitution after GT. Immunological reconstitution after GT with age-matched reference values adjusted for each patient, CD3 + (Panel A), CD19 + (Panel B), CD56 + (Panel C) and CD15 + (Panel D). Age-matched reference values are indicated by the grey lines (mean values, dashed lines, lower and upper values, dotted lines).

**Extended Data Fig. 4:**
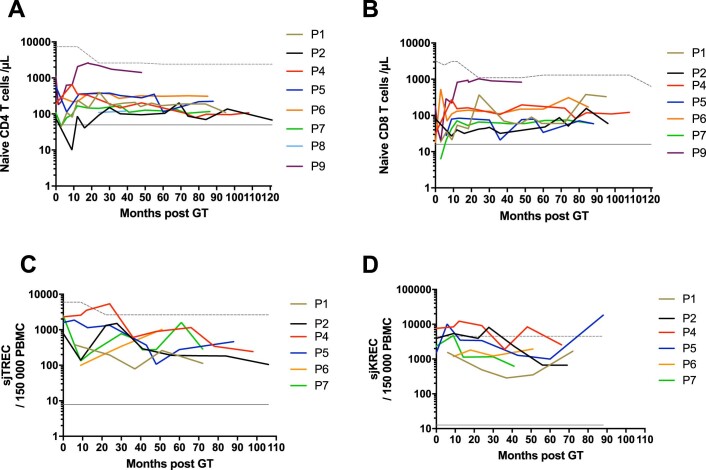
Immunological reconstitution after GT. Immunological reconstitution after GT of naive CD4 + T cells, CD4 + CD45RA + CD31 + cells (Panel A), naive CD8 + T cells, CD8 + CD45RA + CCR7 + cells (Panel B), Tcell receptor excision circles (sjTRECs) (Panel C) and immunoglobulin kappa-deleting recombination excision circles (sjKREC) (Panel D). Age-matched reference values are indicated by the grey lines (solid line: lower value; dashed line: upper value).

In all of the treated patients, the T cell counts increased over time and reached normal age-matched reference values (Fig. 1 and Extended Data Fig. 3). A gradual enrichment in WASp-positive CD3^+^ T cells demonstrated the proliferative and functional advantage of gene-corrected cells over non-corrected cells (Fig. 1). In all of the patients, the naive T cell and signal joint T cell receptor excision circle (sjTREC) counts were similar to those observed in age-matched controls, highlighting active thymopoiesis (Extended Data Fig. 4a–c). Using next-generation sequencing (NGS), the polyclonal T cell antigen receptor-beta (TCRβ) repertoire was analyzed in seven patients 24 and 48 months after gene therapy (Fig. 2). The repertoire diversity was similar to that observed in healthy donors, except for in the three patients (patients 1, 6 and 8) who received the lowest dose of gene-corrected HSPCs (Fig. 2a). The TCRβ repertoire diversity was correlated with the number of corrected HSPCs per kilogram infused (Fig. 2b); it was stable, and no clonal expansion was detected (Fig. 2c).

**Fig. 3:**
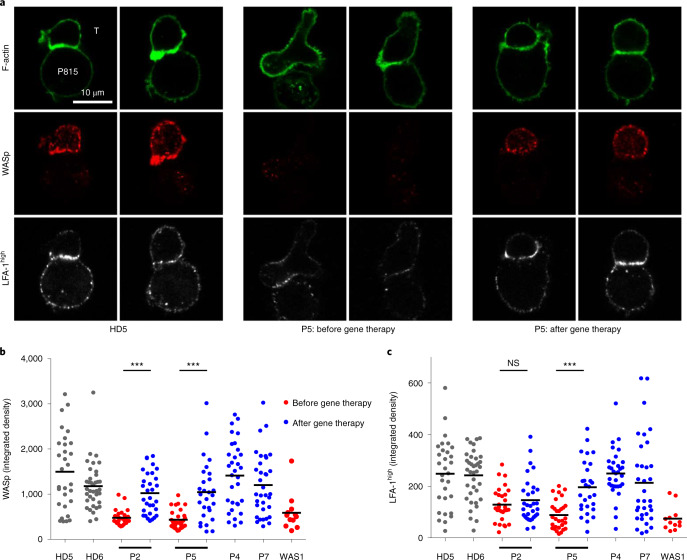
Restoration of T cell immune synapse assembly after gene therapy. **a**, Representative confocal microscopy images of conjugates between the indicated T cells (T, upper cells) and monoclonal anti-CD3 antibody-coated P815 cells (P815, lower cells), after staining for F-actin, WASp and high-affinity LFA-1. The T cells from P5 were collected 55 months after gene therapy; comparable data were collected from additional patients before and after gene therapy (P2) or after gene therapy (P4 and P7). The data were reproduced in two independent experiments (including cell preparation, staining and image acquisition). **b**, Integrated fluorescence density for WASp in T cells forming conjugates with monoclonal anti-CD3 antibody-coated P815 cells in patients with WAS before and after gene therapy (43, 55, 48 and 41 months afterwards for P2, P5, P4 and P7, respectively), in HDs (HD5 and HD6), and in one non-treated patient with WAS (WAS 1). **c**, Integrated fluorescence density for high-affinity LFA-1 in the same cells as in **b**. Overall, 28–37 conjugates per sample were analyzed. Similar results were obtained in another independent experiment. Statistical significance was assessed by unpaired *t*-test (two-tailed) (***P < 0.0001); NS, not significant (*P* = 0.3866). Black horizontal lines in **b** and **c** represent the mean.

To gain further insights into the functional recovery of the T cell compartment, we characterized the immune synapse in expanded T cells from four patients after gene therapy and compared these post-gene therapy data with the pre-gene therapy data, when available (Fig. 3). As shown for a representative patient in Fig. 3, confocal microscopy assessment of T cells forming conjugates with monoclonal anti-CD3 antibody-coated P815 cells showed that the immune synapse assembly was aberrant before gene therapy. The WASp-deficient T cells were elongated, lacked F-actin enrichment at the contact area and featured various types of actin-rich protrusions outside the contact area. Furthermore, the intensity of high-affinity lymphocyte function-associated antigen-1 (LFA-1) staining was lower than for control cells. After gene therapy, the T cells expressed WASp at levels similar to those in healthy donors. WASp was located in the cytoplasm, with enrichment in the synapse (as seen in healthy donors). The post-gene therapy T cells had normalized immune synapse features, including the cell shape, F-actin enrichment and intensity of high-affinity LFA-1 (except in patient 1’s T cells, in which the intensity of high-affinity LFA-1 remained low).

Overall, T cell compartment analysis documented robust immune reconstitution after gene therapy, which was associated with sustained clinical benefit.

### B cell immune reconstitution after gene therapy

**Fig. 4:**
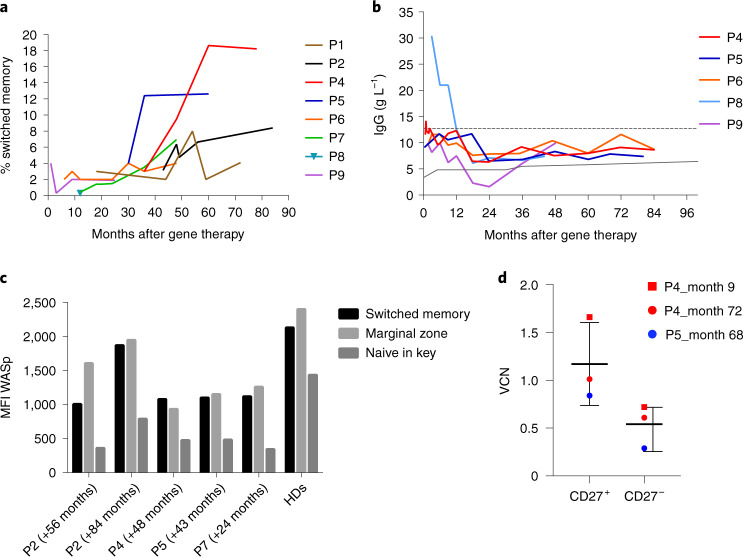
B cell reconstitution after gene therapy. **a**, Switched memory B cell subsets over time. **b**, IgG production in patients who discontinued intravenous immunoglobulin replacement therapy. Age-matched reference values are indicated by the gray lines (solid line, lower value; dashed line, upper value). **c**, WASp expression (mean fluorescence intensity (MFI) in arbitrary units) in switched memory, marginal zone and naive B cell subsets in patients (P) and healthy donors (HD7–HD11). **d**, The VCN in memory B cell (CD27^+^) and naive B cell (CD27^−^) subsets (*n* = 3 biologically independent samples). Data are presented as the mean ± s.e.m.

**Extended Data Fig. 5:**
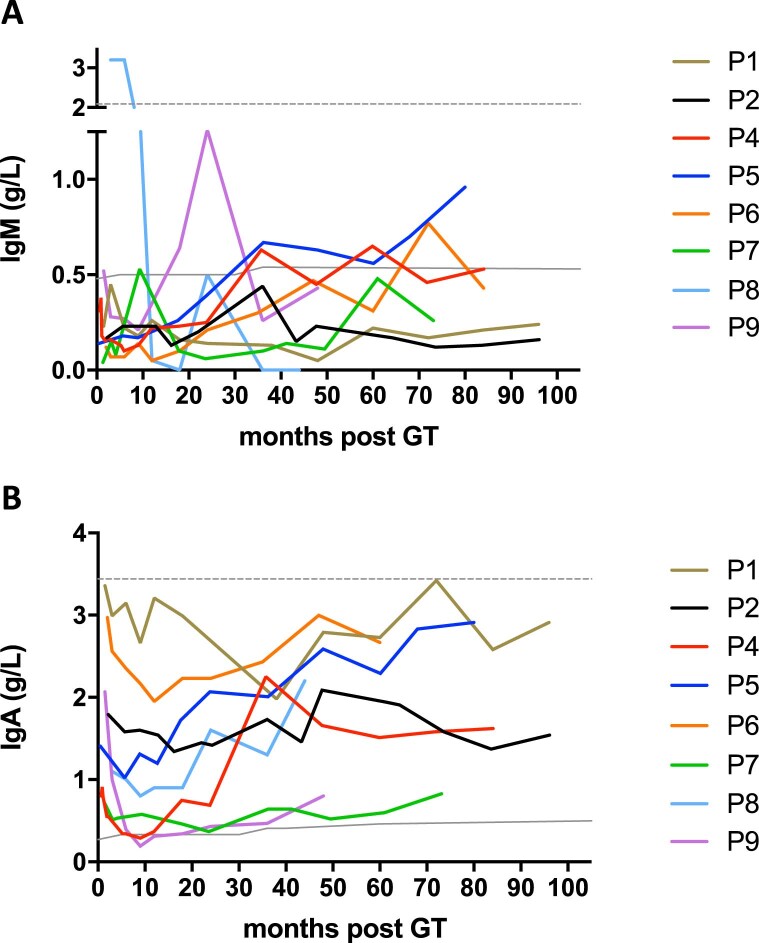
Immunoglobulin reconstitution after GT. Immunoglobulin (Ig) reconstitution after GT. Panel A, IgM production over time; Panel B, IgA production over time. Age-matched reference values are indicated by the grey lines (solid line: lower value; dashed line: upper value).

The B cell count increased progressively over time after gene therapy and reached age-matched reference values in all of the patients at last follow-up (Fig. 1). An enrichment in WASp-positive B cells was observed at the last follow-up, relative to baseline. This enrichment demonstrated the advantage of gene-corrected B cells over non-corrected cells, albeit to a lesser extent than for T cells. Accordingly, kappa-deleting recombination excision circle (KREC) counts, indicative of B cell neogenesis, were in the normal range (Extended Data Fig. 4d). Phenotype analysis showed progressive normalization of the distribution of different subsets in the B cell compartment, with an increase in the switched memory B cell subset in particular (Fig. 4a). No expansion of the CD21^low^CD38^low^ B cell subset (including autoreactive B cells) was observed when compared with in-house reference values from healthy donors. The immunoglobulin production level increased progressively over time: the IgA levels reached age-matched reference values in all treated patients, although IgM levels remained below normal in four patients (Extended Data Fig. 5). At the time of our previous report^20^, only two patients (patients 4 and 5) had been able to discontinue immunoglobulin replacement therapy. Since then, three additional patients (patients 6, 8 and 9) have discontinued immunoglobulin replacement and had normal levels of IgG production (Fig. 4b). When available, the presence of protective levels of post-vaccination antibodies was highlighted (patients 4, 5 and 8; Supplementary Table 2). Patient 2 remains on immunoglobulin replacement therapy for the sequela of vasculitis, as described above.

For four patients, we were able to analyze WASp expression in the B cell subsets after gene therapy and compare it with the level in healthy donors (Fig. 4c). All of the patients had a greater proportion of WASp-expressing cells in the B cell memory subsets (including switched (CD19^+^CD27^+^IgD^−^) and marginal zone (CD19^+^CD27^+^IgD^+^) B cells) than in naive B cells (CD19^+^CD27^−^IgD^+^); this was confirmed by a greater mean fluorescence intensity for WASp in the memory subsets than in the naive cells. These patterns of WASp expression in B cell subsets were also observed in healthy donors (*n* = 5) analyzed concomitantly. To further characterize these subsets, we analyzed the VCN in sorted memory (CD19^+^CD27^+^) and naive (CD19^+^CD27^−^) B cells. We observed significantly greater gene marking in memory B cells than in naive B cells (Fig. 4d). Furthermore, we compared the transduction level in subpopulations (when available) sorted concomitantly from peripheral blood and from bone marrow samples after gene therapy. We observed higher VCN levels in B cells from peripheral blood than in B cells from bone marrow, which suggests that gene-corrected B cells have a selective advantage after egress from the bone marrow (Extended Data Fig. 2a).

To investigate laboratory variables related to autoimmune features, samples collected before and after gene therapy were compared with regard to a panel of circulating autoantibodies. Before gene therapy, anti-nuclear antibodies (ANAs) were detected in all of the patients tested. Remarkably, the ANA titers after gene therapy were null or insignificant. Patient 2, who initially presented with very severe lower extremity vasculitis, presented with anti-RNP, anti-SmB, anti-SSA Ro and anti-SSB antibodies before but not after gene therapy (Supplementary Table 3). Circulating and platelet-bound antiplatelet auto- and alloantibodies were studied separately (only after gene therapy) and were detected in patients 2, 4 and 7 (Supplementary Table 4).

Taken as a whole, these data suggest that gene therapy is able to correct the B cell compartment in WAS, with evidence for a selective advantage over time for genetically corrected B cells.

### Platelet homeostasis and function after gene therapy

**Fig. 5:**
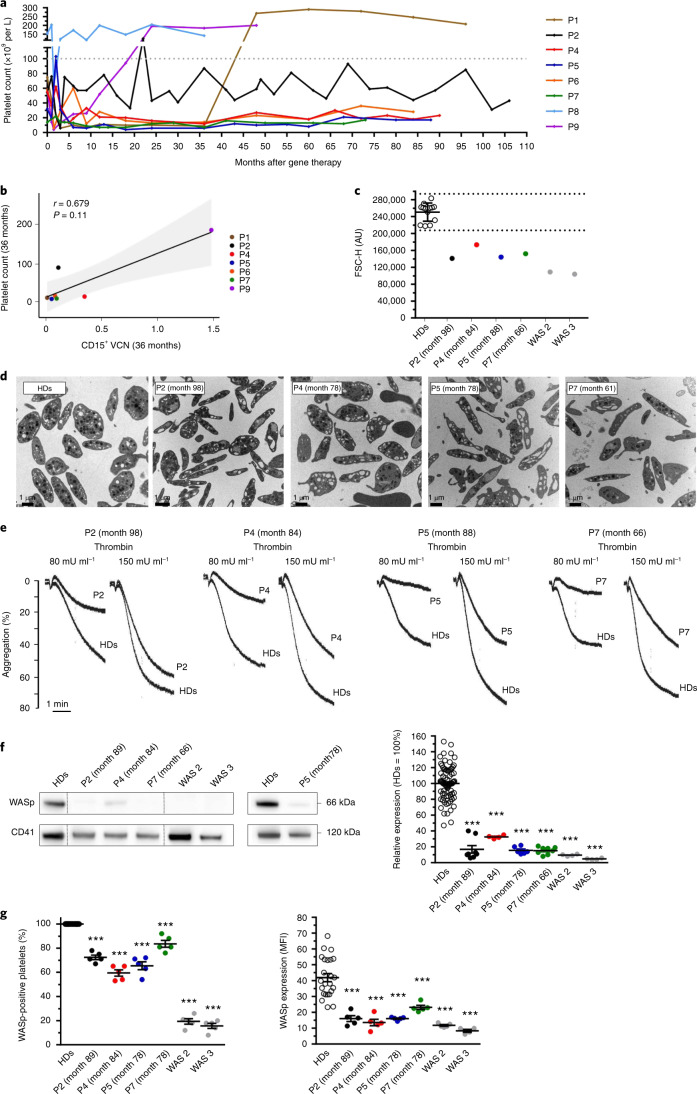
Hemostasis and platelet profile after gene therapy. **a**, Change over time in the platelet count for each patient after gene therapy. **b**, The Spearman correlation between platelet count and the level of gene marking (VCN) in CD15^+^ neutrophils after 3 years of follow-up for each patient (apart from P8, for whom the platelet count was corrected following splenectomy before gene therapy). The *P* value was calculated using the two-sided Spearman’s rank correlation test, and *r* is Spearman’s rank correlation coefficient. A regression line is represented in black and the 95% confidence interval is shown in gray. **c**, Platelet size, evaluated using flow cytometry. Each dot represents the mean size of washed platelets from healthy donors (HDs; *n* = 13), gene therapy-treated patients (P) and non-treated patients with WAS (WAS 2 and WAS 3). The results are expressed as the mean forward scatter height (FSC-H) ± s.d. and the dotted lines indicate the normal range (mean ± 2 s.d.). AU, arbitrary units. **d**, Platelet ultrastructure, which was analyzed once for each patient using transmission electron microscopy. **e**, The thrombin-induced aggregation of washed platelets was evaluated once for each patient (P) and healthy controls (HDs). **f**, WASp expression in patients was evaluated by western blotting. Dotted lines indicate that the samples were derived from the same gel but are non-contiguous. The graph shows the mean of WASp expression ± s.e.m. after normalization against CD41 expression from several independent experiments (HDs, *n* = 82; P4, WAS 2, WAS 3, *n* = 4; P5, *n* = 6; and P2, P7, *n* = 8). **g**, WASp expression was evaluated by immunofluorescence in platelets spread onto a fibrinogen matrix after activation with 20 µM ADP. The dot plot on the left shows the mean of the percentage ± s.e.m. of platelets expressing WASp, and the dot plot on the right shows the mean of the WASp expression (determined from the MFI per platelet) ± s.e.m. for five fields of view, which represent a total number of analyzed platelets of 816 for HDs, 170 for P2, 196 for P4, 175 for P5, 145 for P7, 158 for WAS 2 and 168 for WAS 3. In **f** and **g** statistical significance was determined using one-way analysis of variance with Dunnett’s post test for multiple comparisons. In **f** (right) and **g** (left), the exact *P* value was *P* < 0.0001 for all of the patients, and in **g** (right) the exact *P* value was *P* < 0.0001 for P2, P4, P5, WAS 2, WAS 3 and *P* = 0.0007 for P7. In **c**–**g**, all HDs were different donors, except in **g** (left and right) where HDs were the same.

**Extended Data Fig. 6:**
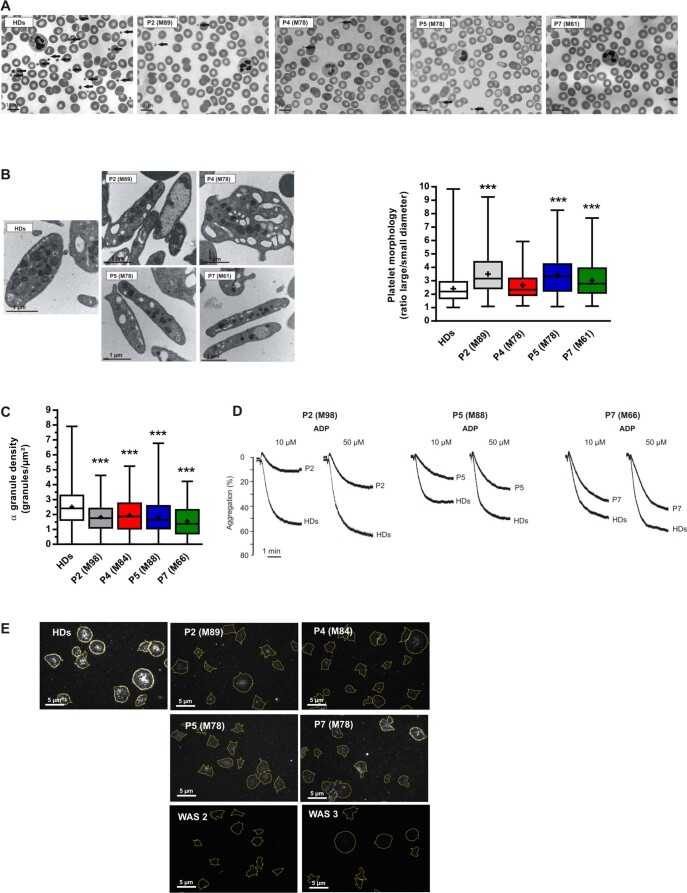
Platelet analyses. Panel A, Images show blood smears performed once for each GT-treated patients (P) or healthy donors (HDs) after May-Grünwald Giemsa staining. Arrows indicate the platelets. Panel B, TEM analysis of platelet ultrastructure in each patient (P). The box-and-whisker plots show the platelet shape defined as the ratio between the largest diameter and the smallest diameter derived from the TEM images performed once for each patient. Whiskers represent the upper and lower extreme values, the box correspond to the interquartile range, the center line is the median and the cross indicates the mean of 100 platelets for patients and 826 platelets for HDs. Statistical significance was determined in a one-way ANOVA with the Sidak post-test for multiple comparisons. The exact p value was p < 0.0001 for P2, P5, P7 and p = 0.1484 for P4. Panel C, the α-granule density for each patient was quantified by TEM and the results were shown in box-and-whisker plots. Whiskers represent the upper and lower extreme values, the box correspond to the interquartile range, the center line is the median and the cross indicates the mean of 100 platelets for patients and 970 for HDs. Statistical significance was determined in a one-way ANOVA with the Sidak post-test for multiple comparisons. The exact p value was p < 0.0001 for all P2, P5, P7 and p = 0.0002 for P4. Panel D, Aggregation of washed platelets induced by ADP (10 or 50 µM) was evaluated once for P2, P5 and P7. Panel E, WASp expression was evaluated by immunofluorescence in platelets spread onto a fibrinogen matrix after activation with 20 µM ADP. WASp was detected using a specific antibody. The outlines of spread platelets (represented by the yellow lines) were identified by detecting F-actin with fluorescently labeled phalloidin.

**Extended Data Fig. 7:**
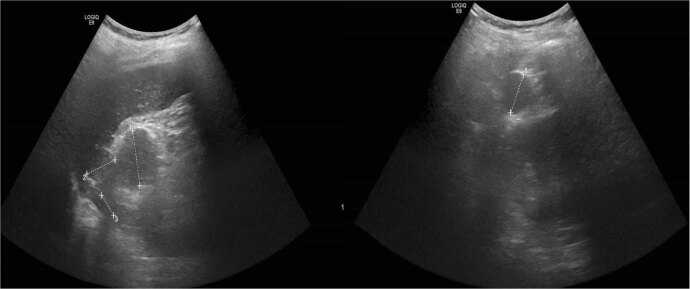
Ultrasound assessment of P2. Ultrasound assessment of P2, showing three accessory spleens (measuring up to 3 cm in diameter) in the right hypochondrium.

After gene therapy, platelet count increased over time and reached values of more than 140,000 platelets per µl at last follow-up in three patients (Fig. 5). These include patients 1 and 8, in whom thrombocytopenia was stably corrected after splenectomy (at 36 months after gene therapy and before gene therapy, respectively), and patient 9 (the patient with the highest engraftment of gene-corrected myeloid cells). For the other patients, the platelet count remained significantly below the normal range: from 20,000 to 50,000 platelets per µl for patients 2, 4 and 6 and below 20,000 platelets per µl for patients 5 and 7 (Fig. 5a and Extended Data Fig. 6a). Patient 2 had a splenectomy before gene therapy at the age of 8 years. This led to a temporary increase in the platelet count (up to 80,000 platelets per µl), followed by fluctuations and the observation of accessory spleen on ultrasound (Extended Data Fig. 7). For all of the patients, the platelet count 3 years after gene therapy tended to correlate with the level of gene marking in CD15^+^ cells (Fig. 5b). Patients (including those with below-normal platelet counts) did not present with spontaneous bleeding after gene therapy and no longer required platelet transfusions. Patients 4 and 7 received preventive platelet transfusions prior to scheduled surgery (7 years and a few months after gene therapy, respectively). Following a family decision, patient 5 started treatment with eltrombopag, a thrombopoietin receptor agonist (TRA), 6 years after gene therapy, without significant improvement in the platelet count. Six and a half years after gene therapy, patient 7 presented with a drop in the platelet count (5,000 platelets per µl) and developed cutaneous and mucosal ecchymosis. Treatment with the TRA romiplostim was initiated and led to a moderate increase in the platelet count (up to 34,000 platelets per µl).

To better characterize the platelets after gene therapy, a detailed functional and phenotypic analysis was performed on four patients from the same center (Necker-Enfants Malades Hospital). Platelet size was evaluated using flow cytometry, which is more accurate than automated blood cell counting, especially for small platelets. The results for the patients treated with gene therapy were compared with those for healthy donors and non-treated patients with WAS. We found that the platelets of the patients treated with gene therapy were larger than those of the non-treated patients with WAS but were still smaller than those of healthy donors (Fig. 5c). Moreover, transmission electron microscopy ultrastructure analysis showed that the platelets of the patients treated with gene therapy were elongated; this contrasted with the disk-like shape of resting platelets from healthy donors (Fig. 5d and Extended Data Fig. 6b). The α-granule density was also slightly lower than normal, due to the smaller cell size (Extended Data Fig. 6c). To further evaluate hemostasis and platelet function after gene therapy, we studied the aggregation of washed platelets (Fig. 5e). At a low thrombin concentration (80 mU ml^−1^), all of the patients had severe defects in platelet aggregation compared with healthy donors. Aggregation was less abnormal at a higher thrombin concentration (150 mU ml^−1^), which suggests a partially functional response to agonist. Similar results were also observed after activation with adenosine diphosphate (ADP) (Extended Data Fig. 6d). To further evaluate the degree of platelet correction, WASp expression was evaluated using western blotting (Fig. 5f). All of the patients treated with gene therapy had suboptimal WASp expression (15–32% of that measured in healthy donors). The level of WASp expression after gene therapy was nevertheless higher than that observed in non-treated patients with WAS (5–10% of healthy donor values).

Next, we used an immunofluorescence assay to quantify WASp expression (Extended Data Fig. 6e) in individual platelets and to estimate the percentage of WASp-positive platelets and the level of WASp expression per platelet (Fig. 5g). The proportion of WASp-positive platelets ranged from 60% to 84% in the patients treated with gene therapy and from 16% to 19% in non-treated patients with WAS (Fig. 5g, left). It must be noted, however, that one of the two non-treated patients harbored a mutation resulting in residual WASp expression. However, the level of WASp expression per platelet in the gene therapy-treated patients was significantly lower (by 45–67%, *P* < 0.001) than in healthy donors (Fig. 5g, right). This indicates that, although a relatively high proportion of platelets re-expressed WASp after gene therapy, the level of WASp expression in individual platelets remained suboptimal. Last, antiplatelet antibodies were found in patients 2, 4 and 7 after gene therapy, as mentioned above.

Taken as a whole, these platelet measurements show that the gene therapy procedure only partially corrected the platelet compartment. However, this suboptimal recovery benefited the patients and was sufficient to prevent episodes of spontaneous hemorrhage.

### Integration site and replication-competent lentivirus analyses

**Extended Data Fig. 8:**
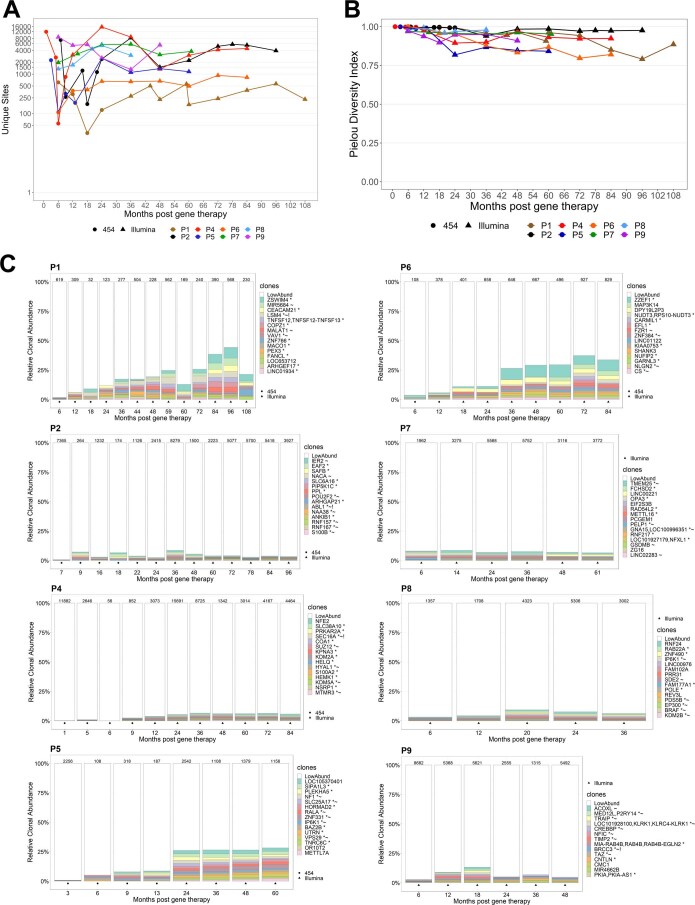
Integration site (IS) analyses. Panel A, The number of unique ISs detected in PBMCs at various points (in months) after gene therapy. Most of the samples were analyzed by sonic shearing in Illumina sequencing experiments (triangle), except that the early samples were analyzed using restriction endonucleases in 454 sequencing experiments (circle). Panel B, The Pielou diversity index for the IS population in PBMCs for the different patients. Panel C, A stacked bar representation showing the relative clone size (sonic abundance) of the TOP15 integration site clones detected over time for each patient.

Vector integration site analysis of peripheral blood mononuclear cells (PBMCs) from the eight patients revealed a diversity of integration sites, with between 169 and 15,691 different sites (as analyzed using Illumina sequencing; Extended Data Fig. 8a). We observed high, time-stable values of the Pielou diversity index (a normalized version of the Shannon diversity index) for integration sites in the various patients^25,26^ (Extended Data Fig. 8b), reflecting successful polyclonal gene correction. An analysis of the most abundant clones over time also highlighted strong, stable polyclonal reconstitution (Extended Data Fig. 8c), with no concerning clonal expansion. The monitoring of replication-competent lentivirus occurrence that was conducted as part of the human immunodeficiency virus (HIV) mRNA evaluation has been consistently negative at all time points tested in all of the present patients.

## Discussion

We have previously reported preliminary data on seven patients with WAS who were treated with gene therapy and whose follow-up ranged from 9 to 42 months^20^. In this updated report, we provide a comprehensive long-term analysis of data from the longest follow-up reported to date, with a median of 7.6 years (range, 4–9 years), and include two additional patients treated since the last report. The present study confirms our earlier results and provides additional evidence of the long-term efficacy of gene therapy for WAS, as well as the safety track record. Here, sustained multilineage engraftment of gene-modified cells was observed in the peripheral blood and the bone marrow. In turn, this led to increasing expression of WASp in the patients’ cells and clinical resolution of severe eczema and susceptibility to recurrent infections. In line with these results, T cell function was restored, as shown by the recovery of immune synapse assembly and normal naive T cell count and functions after gene therapy. The T cell compartment was reconstituted in the patient treated at the age of 30 years, suggesting that gene therapy for WAS is also a treatment option in adult patients^22^, despite the continuing risk associated with splenectomy^27^.

In parallel with the robustness of T cell reconstitution, a normalized B cell compartment was observed, in line with previous studies^28,29^, as shown in particular by the increasing level of WASp^+^ switched memory B cells over time and the age-matched level of KRECs. Five patients were able to discontinue prophylactic antibiotic treatment and immunoglobulin replacement therapy while achieving normal post-vaccination antibody titers. The level of gene marking was greater in lymphoid than in myeloid cells. This is consistent with the strong proliferative and/or survival advantage conferred by WASp expression in the lymphoid compartment, as predicted by earlier observations in mice and in patient cells^30,31^. This was also reported in the WAS gene therapy trial conducted in Milan with the same vector^21^.

No graft failure or treatment-related adverse events were observed after gene therapy. Vector integration site analyses showed that the insertion profile was safe and diverse, with no concerning clonal expansion.

In the present study, we focused on two major concerns in patients with WAS: thrombocytopenia and autoimmunity. The mechanisms of thrombocytopenia in WAS include defective central thrombocytopoiesis and increased peripheral destruction^32–34^. A recent study has highlighted functional and phenotypic abnormalities and an altered proteomic profile in WASp-deficient platelets^34^. This senescent profile accelerates the peripheral destruction of platelets. After gene therapy, the patients had a marked decrease in the frequency and severity of spontaneous bleeding episodes. Platelet count increased over time and reached more than 140,000 platelets per µl in three patients (that is, two splenectomized patients and the patient with the strongest engraftment of gene-corrected myeloid cells). Platelet count in the other patients remained subnormal, perhaps because of the low WASp expression in this lineage or the residual autoimmunity in some patients, as recently described in a conditional mouse model^33^. Given that the gene marking level in the myeloid lineage reflects the engraftment of corrected HSCs in the bone marrow, one can propose that the initial quantity of HSCs infused and the level of gene marking in the cell product influence the recovery of platelet count. A higher level of gene correction might also improve the migration of true stem cells into the bone marrow. The low degree of myeloid chimerism, despite adequate VCN and HSC counts in the infused drug product, might also be due to the defective migration of WASp^low^ HSCs described previously^35^.

After gene therapy, a few autoimmune manifestations were observed: persistence of lower extremity vasculitis (in patient 2), new nephrotic syndrome occurrence (in patient 9) and presence of antiplatelet antibodies (in patients 2, 4 and 7). The level of circulating autoantibodies detected before gene therapy (including ANA and vasculitis-related autoantibodies) was normalized after treatment. Further improvement in autoimmune features after gene therapy might result from the inclusion of an anti-CD20 agent in the conditioning regimen.

The main difference with the results from the Milan trial^19–21^ concerned platelet count. The count was below the normal range in both studies but lower in the present patients. This disparity might be due to differences in the severity score and age of the treated patients and in the VCN of the infused drug product. Disease prior to gene therapy was more severe in the present trial than in the Milan trial, as evidenced by the median Zhu score (5 versus 4, respectively). Moreover, the present patients were older at the time of gene therapy than those in the Milan trial (median age of 5.25 years versus 2.2 years, respectively). Last, the median VCN was lower in the present trial than in the Milan trial (0.9 versus 2.4, respectively).

Further lentiviral vector optimization (with a stronger promoter, for example) has been considered as a possible means of enhancing WASp levels in platelets^36–38^. Alternatively, enhancement of the ex vivo transduction procedure might usefully result in higher levels of gene correction of WAS HSPCs in terms of both cell frequency and the VCN.

In conclusion, gene therapy is a safe, efficacious treatment for patients with WAS who lack a suitable HSCT donor. More efficacious and more reliable transduction protocols and conditioning regimens are likely to improve outcomes further, particularly with regard to platelet recovery, for which the advantages of intrinsic correction are less apparent.

## Methods

### Study design and patient enrollment

We have previously reported on a non-randomized, open-label, phase I/II clinical study (based on a lentiviral gene therapy vector) involving seven pediatric patients with severe WAS. Details of the gene therapy study design and procedures have been reported previously^20^. The patients were treated at Necker-Enfants Malades Hospital (Paris, France; ClinicalTrials.gov identifier NCT01347346) and at Great Ormond Street Hospital (London, UK; ClinicalTrials.gov identifier NCT01347242). Patients were enrolled in the Long Term Safety Follow up of Haematopoietic Stem Cell Gene Therapy for the Wiskott–Aldrich Syndrome (WASFUP) study (ClinicalTrials.gov identifier NCT02333760), which was designed to follow patients for an additional 13 years after the initial gene therapy study. The WASFUP study protocol was approved by the UK and French drug regulatory agencies and the appropriate investigational review boards, such as the Gene Therapy Advisory Committee in the United Kingdom and the Comité de Protection des Personnes in France. A CONSORT flow diagram detailing enrollment of the patients is available in the Supplementary Information. Data were collected manually and transferred to the Altizem Society for data management through Clintrial software.

The primary endpoint for the initial WAS trials (NCT01347346 and NCT01347242) was to assess the efficacy of HSC gene therapy in patients with WAS, based on clinical improvement in at least one of the following parameters (depending on the patient symptom profile at study entry): eczema status, the frequency and severity of infections, bruising and bleeding episodes, and autoimmune disorders and the number of disease-related days of hospitalization. The thee secondary objectives were to assess the safety of HSC gene therapy in patients with WAS, the efficacy of HSC gene therapy for the progression of microthrombocytopenia and the need for its treatment, and the efficacy for other hematological variables, including WASp expression and reconstitution of humoral and cell-mediated immunity.

The eligibility criteria were male sex (any age); severe WAS (clinical score 3–5) or absence of WASp in PBMCs determined on western blotting and flow cytometry; molecular confirmation using *WAS* gene DNA sequencing; lack of HLA-genotypically identical bone marrow or 10/10 or 9/10 antigen HLA-matched unrelated donor or HLA-matched cord blood after a 3-month search; signed informed consent or assent by the parent, guardian or patient; willingness to return for follow-up (only for patients who have received previous allogenic HSCT); failed allogenic HSCT; and contraindication to repeat transplantation.

The exclusion criteria were HLA-genotypically identical bone marrow; 10/10 or 9/10 antigen HLA-matched unrelated donor or HLA-matched cord blood; contraindication to leukapheresis; contraindication to bone marrow harvest; contraindication to conditioning medication; and HIV positivity.

The six primary endpoints for the long-term safety follow-up trial (NCT02333760) were the incidence and type of serious adverse events and, more specifically, the incidence and nature of delayed events (such as malignancies, hematologic events, autoimmune events and mortality continuously for the duration of the post-gene therapy follow‐up study); the safety of the gene therapy procedure for gene transfer analysis at yearly post-gene therapy visits (lentiviral integration sites in different cell subpopulations, quantification of the VCN in sorted cell populations using real-time quantitative PCR); the safety of the gene therapy procedure with regard to the absence of replication-competent lentiviruses (RCLs) at yearly post-gene therapy visits; the change in medical conditions; the key medical events related to WAS (eczema status, infections, bleeding symptoms, autoimmune manifestation); and the hematological and cell-mediated and humoral immunity reconstitution. The three secondary endpoints were change in the need for associated treatments at the yearly post-gene therapy visit (immunoglobulin replacement therapy, antibacterial, antifungal and antiviral drugs, transfusions etc.); the representation of TCR families using PCRs, TREC assays and the TCR Vβ panel after gene therapy; and bone marrow integrity (optional) after gene therapy via bone marrow aspiration. The WASFUP study (NCT02333760) was designed as a longitudinal descriptive study without interim analysis. It has included nine eligible and consenting patients between October 2014 and November 2019. Considering the duration of the study (13 years), an intermediate evaluation of the majority of primary and secondary endpoints (delayed events, key medical events, biological parameters of vector expression and insertion, RCL, platelet and immune reconstitution), as well as additional exploratory investigations on T cells and platelets, was conducted for the first eight patients having at least 4 years of follow-up after gene therapy. These eight patients described here were included in the WASFUP study between 29 October 2014 and 13 December 2015. Some prespecified biological and medical parameters of the WASFUP study were not reported, such as duration of hospital stay, humoral or cellular responses to antigens, and bone marrow exploration, given that the clinical study is still ongoing. The two inclusion criteria were enrollment in the initial phase I/II WAS trial conducted in France and the United Kingdom and signed informed consent by the parents, guardians or patients. The only exclusion criterion was unwillingness in parents, guardians or patients to return for the follow-up study period.

Written informed consent or assent was obtained after the benefits and risks of the trial had been explained to the patients or their parents or legal guardians. The study was carried out in accordance with the tenets of the Declaration of Helsinki.

Patients were included in the present study if they had at least 4 years of follow-up. One patient (patient 3) died 7 months after gene therapy from pre-existing complications of infection, as reported previously^20^, and was therefore not included in the present study. Since the initial report, two additional patients treated at Great Ormond Street Hospital have been followed for at least 4 years after gene therapy and so were included in the present study (patients 8 and 9). Data were obtained from blood samples provided by patients with WAS after gene therapy (patients 1–9) at routine hospital visits and were compared with data from blood samples from non-treated patients with WAS (WAS 1–3) and adult healthy donors (healthy donors 1–11). Blood samples from healthy individuals were obtained from the French Blood Establishment (Etablissement Français du Sang (EFS); ref: C CPSL UNT-N°18/EFS/031, and collection DC-2015-2488 approved by the ethics board Comité de Protection des Personnes CPP Sud Ouest et Outre-Mer II (France)) and from adult healthy volunteers upon informed consent, in accordance with the tenets of the Declaration of Helsinki; the study participants were informed about the anonymous use of their personal data. Patient WAS 1 corresponds to patient WAS 2 (male, 5 years) in the 2002 study by Dupré et al.^39^. This patient carries a 2-nucleotide deletion (AG) in exon 4 (position 484–485) of the *WAS* gene resulting in a stop codon (codon 167). Patients WAS 2 and WAS 3 are currently followed at Necker-Enfants Malades Hospital in Paris; sampling was obtained during regular visits at the hospital, upon informed consent. Peripheral blood samples from these patients were obtained in accordance with the tenets of the Declaration of Helsinki (including informed consent). Published age-matched reference values were used for lymphoid subsets^40^, neutrophils^41^ and immunoglobulins^42^. Internal age-matched reference values were generated and used for sjTRECs and sjKRECs (pediatric, *n* = 20; adult, *n* = 199). The CD21^low^CD38^low^ B cell subset data for patients after gene therapy were compared with in-house reference values from healthy controls (*n* = 30) analyzed with the same gating strategy. The diagnosis of WAS was confirmed by genetic testing.

### Outcomes

Since our initial report, we have regularly collected data on a range of clinical and laboratory variables: immune reconstitution, the VCN and WASp expression in sorted myeloid and lymphoid populations, and integration site profiles. T cell correction was evaluated using real-time quantitative PCR assays of sjTRECs, and the TCR repertoire was evaluated using NGS. In four patients, immune synapse assembly was studied before and after gene therapy. Platelet function and phenotype were comprehensively characterized for four gene therapy patients, non-treated patients with WAS and healthy donors. We recorded changes over time in B cell count and function, including subpopulation phenotype, WASp expression in B cell subsets, VCN in the memory (CD19^+^CD27^+^) and naive (CD19^+^CD27^−^) compartments, KRECs, immunoglobulin production and post-vaccination titer. Patients were tested for a panel of autoantibodies (including antiplatelet antibodies) before and after gene therapy, when possible. Age-matched reference values were added for the different immunological parameters, based on pediatric reference ranges^40,41^.

### Isolation of mononuclear cells

In line with the trial protocol, peripheral blood was sampled regularly during follow-up, while bone marrow was sampled once after gene therapy (unless required more frequently for medical reasons). Mononuclear cells were isolated from peripheral blood or bone marrow using standard Ficoll gradient separation. Lymphocyte subpopulations were studied using fluorochrome-coupled antibodies (BD Bioscience, Miltenyi Biotec), and the absolute lymphocyte count was determined using Trucount Tubes (BD Bioscience), as described previously^20^.

### Immunophenotyping and sorting of hematopoietic subpopulations

Cells were stained with specific, directly labeled monoclonal antibodies, according to the manufacturer’s instructions and as described previously^20^. An eight-color FACSCanto II cell analyzer and FACSAria II cell sorter (BD Biosciences) were used for flow cytometry analysis and cell sorting, respectively, according to the manufacturer’s instructions. Flow cytometry data were analyzed using FlowJo software (TreeStar).

### Proliferation assays

T cell lymphoproliferation was assayed by measuring the incorporation of tritiated thymidine in response to a challenge with phytohemagglutinin (Sigma-Aldrich) or tetanus toxoid (Staten), as described previously^20^.

### Clonogenic assay and DNA extraction

Erythroid burst-forming unit and granulocyte–macrophage colony-forming unit progenitors or precursors were grown in semisolid methylcellulose medium supplemented (or not) with erythropoietin (Methocult H4435 and H4535, respectively; STEMCELL Technologies), according to the manufacturer’s instructions. Single colonies were picked for DNA extraction.

### Gene marking

Gene marking in sorted subpopulations was monitored via VCN analysis, as described previously^20^.

### TREC and KREC analysis

sjTRECs and sjKRECs were quantified using a real-time PCR assay, as described previously^43^. In brief, a 1:200 or 1:2,000 dilution of an initial multiplex PCR amplification was quantified in duplicate for each of the different primer–probe sets on a ViiA7 Real-Time PCR System (Applied Biosystems).

sjTREC and sjKREC counts are given per 150,000 cells (around 1 µg DNA) after normalization against the albumin gene content. Data are expressed as the log_10_ count per 150,000 PBMCs.

### TCR repertoire analysis

To analyze the TCR repertoire, genomic DNA was extracted from blood samples or PBMCs. The TCRβ repertoire was assessed with one-step NGS using 100 ng DNA and EuroClonality-NGS amplicon primers^44^. The DNA was sequenced on an Illumina MiSeq system, using 2 × 250-bp v2 chemistry. Figures were prepared with R software (https://www.R-project.org/). The Shannon diversity index was calculated using the vegan package. Circos plots showing the various V-gene and J-gene combinations were designed using the circlize package.

### Detection of autoantibodies

Serum samples from all participants were screened for ANAs using HEp-2000TM commercial slides (Immunoconcept). HEp-2000TM cells are HEp-2 cells that overexpress Ro60 antigens. Serum samples were diluted 1:80 in PBS and incubated for 25 min at room temperature in a humid chamber. After two washes in PBS, cells were incubated with FITC-conjugated goat anti-human IgG (immunoglobulin heavy and light chains, Immunoconcept) for another 25 min in the dark. After two more washes in PBS, the slides were counterstained with Evans blue and assembled with glycerol and coverslips. Specific antibodies were detected using a multiplex ENA (extractable nuclear antigen) assay on the FIDIS instrument (Theradiag), an anti-DNA ELISA (DiaSorin), a Farr assay (Trinity Biotech) and/or an anti-nucleosome ELISA (Werfen). Samples were also screened for anti-neutrophil cytoplasmic antibodies (ANCAs) using commercial NOVA LiteH ANCA ethanol or formalin slides. Thirty microliters of a sample diluted 1:20 was spotted on each well, incubated for 25 min and washed with PBS buffer (Immunoconcept). Cells were subsequently incubated with FITC-conjugated goat anti-human immunoglobulin for 25 min in the dark. After two washes in PBS, slides were counterstained with Evans blue and assembled with glycerol and coverslips. Indirect immunofluorescence screening for anti-tissue antibodies on triple rodent tissue (stomach, liver, kidney; Biosystems) was performed with IgG antibodies against smooth muscle, mitochondria, LKM1, parietal cells, LC1, SLA and intrinsic factor. Serum samples were diluted 20-fold in PBS and incubated for 30 min at room temperature in a moist chamber. After two washes in PBS, the slides were incubated with FITC-conjugated goat anti-human IgG, IgM and IgA antibodies. Slides were examined under a fluorescence microscope. To minimize subjective bias, two observers had to agree on the results of the ANCA assay, the ANA assay and the indirect immunofluorescence screening for anti-tissue antibodies.

### Evaluation of T cell immune synapse assembly

T cells were expanded by stimulation of PBMCs with irradiated PBMCs and Epstein–Barr virus-immortalized B cells (JY cells), interleukin (IL)-2 (100 U ml^−1^) and IL-15 (5 ng ml^−1^) in RMPI medium containing 5% human serum. As an internal control, a T cell line derived from a previously described patient with WAS (referred to here as WAS 1, corresponding to patient 2 in the report by Dupré et al.^39^) was added to the samples from the gene therapy-treated patients. To rule out selection bias in the in vitro expansion step, we confirmed that the VCN at the time of immune synapse analysis was similar to that measured at sampling (median VCN, 1.29 and 1.15, respectively). Immune synapse assembly was evaluated as described previously^45^ by mixing T cells with anti-CD3 monoclonal antibody (mAb) (10 μg ml^−1^ OKT3, eBioscience)-coated P815 cells and depositing the resulting cell conjugates on poly(l-lysine)-coated slides with preformed reaction wells (Marienfeld). Unlabeled a24 mAb (Biolegend) that binds to LFA-1 in its high-affinity conformation was added at a concentration of 2.5 µg ml^−1^ for 10 min at 37 °C. The conjugates were then fixed, permeabilized and stained with phalloidin-AF488 (Invitrogen) and anti-WASp rabbit mAb (Abcam, ab75830). The anti-LFA-1 and anti-WASp antibodies were then visualized with anti-mouse AF647-coupled and anti-rabbit AF564-coupled secondary antibodies (Invitrogen), respectively. Slides were examined under an LSM-710 confocal microscope (×63/1.4 oil immersion Plan-Apochromat objective, Carl Zeiss). To calculate the integrated density of WASp and high-affinity LFA-1, images of randomly selected T cell:P815 cell conjugates were acquired and then analyzed with ImageJ software.

### Platelet characteristics and function

#### Preparation of washed platelets

Venous blood from healthy donors or patients was collected in 10% ACD/A buffer (75 mM sodium citrate, 44 mM citric acid and 136 mM dextrose, pH 4.5). As described previously^46^, platelets were washed in the presence of apyrase grade VII (100 mU ml^−1^, Sigma-Aldrich) and prostaglandin E_1_ (1 µM, Sigma-Aldrich) to minimize platelet activation. The platelet count was then adjusted to 3 × 10^8^ platelets per ml in Tyrode’s buffer (137 mM NaCl, 2 mM KCl, 0.3 mM NaH_2_PO_4_, 1 mM MgCl_2_, 5.5 mM glucose, 5 mM *N*-(2-hydroxyethyl)piperazine-*N*′-2-ethanesulfonic acid, 12 mM NaHCO_3_ and 2 mM CaCl_2_, pH 7.3).

#### Flow cytometry evaluation of platelet size

Platelet size was evaluated using a BD Accuri C6 flow cytometer (BD Biosciences) by recording the forward scatter parameters.

#### Platelet aggregation

Platelet aggregation was monitored by measuring light transmission through a stirred suspension of washed platelets (3 × 10^8^ platelets per ml) at 37 °C, using a Chrono-Log aggregometer (Chrono-Log), as described previously^47^. Platelet aggregation was triggered by bovine thrombin (Sigma-Aldrich) and ADP.

### Western blotting for WASp expression

Washed platelets (3 × 10^8^ platelets per ml) were lysed in Laemmli sample buffer (10 mM HEPES, 2% SDS, 10% glycerol, 5 mM EDTA). The proteins were reduced by incubation with 25 mM dithiothreitol, separated by electrophoresis using NuPage 4–12% Bis-Tris Protein gels (Invitrogen) and transferred to nitrocellulose membranes, which were incubated with mouse anti-WASp primary antibody (1 µg ml^−1^, clone B-9; Santa Cruz) or mouse anti-CD41 (0.2 µg ml^−1^, clone SZ22, used as a loading control for normalization; Beckman Coulter). Immunoreactive bands were visualized with enhanced chemiluminescence detection reagents (Perbio Science) and a G:BOX Chemi XT16 Image System and were then quantified using Gene Tools v4.03.05.0 (Syngene).

### Immunofluorescence assay of WASp expression

Glass coverslips were coated with 100 μg ml^−1^ human fibrinogen (HYPHEN BioMed SAS) in PBS (0.01 M H_3_PO_4_, 0.154 mM NaCl, pH 7.4 ± 0.2) at 4 °C overnight and then blocked with 5% BSA. Washed platelets (3 × 10^6^) were stimulated with ADP (20 µM, to induce full platelet spreading; Sigma-Aldrich), plated on fibrinogen-coated glass coverslips and incubated for 30 min at 37 °C. After washing, platelets were fixed for 15 min with 4% paraformaldehyde, permeabilized with 0.2% Triton X-100, blocked with 1% BSA and stained with primary mouse anti-WASp antibody (4 μg ml^−1^, clone B-9; Santa Cruz). The primary antibody was detected using a goat anti-mouse IgG Alexa Fluor 555 conjugate (4 µg ml^−1^, Invitrogen), and platelet morphology was assessed by labeling cytoskeletal F-actin with Alexa Fluor 488-phalloidin (0.3 µM, Invitrogen). Last, the coverslips were mounted on slides, and immunofluorescence images were acquired on an epifluorescence microscope (Nikon, Eclipse 600). WASp expression was quantified using ImageJ.

### Transmission electron microscopy

Washed platelets were fixed by incubation for 1 h at room temperature in 1.25% glutaraldehyde in 0.1 M phosphate buffer (pH 7.2), centrifuged for 10 min at 1,100*g* and washed once in 0.1 M phosphate buffer. Platelets were stored in 0.2% glutaraldehyde at 4 °C until processing for standard transmission electron microscopy analysis of platelet morphology, as described previously^48^.

### Detection of antiplatelet autoantibodies and/or alloantibodies

For each patient analyzed, 15 ml blood was collected in EDTA tubes for the detection of autoantibodies bound to platelet glycoprotein complexes. Serologic assays for circulating autoantibodies and/or alloantibodies were performed on 10 ml blood collected in dry tubes. Patients’ antiplatelet antibodies were detected using monoclonal antibody-specific immobilization of platelet antigen technique, according to the manufacturer’s instructions (apDia)^49^.

### Integration site analyses

Vector integration sites were amplified, sequenced and analyzed using the INSPIIRED pipeline, as described previously^50–52^. The Pielou diversity index (a normalized version of the Shannon diversity index) was calculated using the vegan package.

### Statistical analysis

Results are reported as the mean ± s.d. or the median, unless stated otherwise. All statistical analyses were performed using GraphPad Prism6 (GraphPad). Data were analyzed using one-way analysis of variance followed by a post hoc test, as indicated in the figure legends. The threshold for statistical significance was set to *P* < 0.05. The statistical significance of the Spearman correlation between parameters was evaluated using the cor.test function in R software.

### Reporting Summary

Further information on research design is available in the Nature Research Reporting Summary linked to this article.

## Online content

Any methods, additional references, Nature Research reporting summaries, source data, extended data, supplementary information, acknowledgements, peer review information; details of author contributions and competing interests; and statements of data and code availability are available at 10.1038/s41591-021-01641-x.

## Supplementary information

Supplementary Information Supplementary Tables 1–4 and CONSORT flowchart.

Reporting Summary

## Data Availability

Data that support the findings in this study are available from the authors upon agreement of the sponsor (S.A., Genethon). Restriction may apply to the availability of these data before the end of the study as they are part of clinical trials, subject to patient confidentiality, and are not public. Integration site sequence data used in the present study are available in the NCBI Sequence Read Archive (SRA, reference nos. SRP050221 and PRJNA685802). TCR NGS sequence data and RCL analyses are available upon request. Source data are provided with this paper.

## Source data

Fig. 5f Unprocessed western blot.
Fig. 5f Unprocessed western blot.
Fig. 5f Unprocessed western blot.
Fig. 5f Unprocessed western blot.
Fig. 5f Unprocessed western blot.

## Extended data

are available for this paper at 10.1038/s41591-021-01641-x.

## Code availability

Several functions and packages from R software (https://www.R-project.org/) have been used for analyses as mentioned in the specific sections.
