# Author Correction: Long-term safety and efficacy of lentiviral hematopoietic stem/progenitor cell gene therapy for Wiskott–Aldrich syndrome

**DOI:** 10.1038/s41591-022-01985-y

**Published:** 2022-08-09

**Authors:** A. Magnani, M. Semeraro, F. Adam, C. Booth, L. Dupré, E. C. Morris, A. Gabrion, C. Roudaut, D. Borgel, A. Toubert, E. Clave, C. Abdo, G. Gorochov, R. Petermann, M. Guiot, M. Miyara, D. Moshous, E. Magrin, A. Denis, F. Suarez, C. Lagresle, A. M. Roche, J. Everett, A. Trinquand, M. Guisset, J. Xu Bayford, S. Hacein-Bey-Abina, A. Kauskot, R. Elfeky, C. Rivat, S. Abbas, H. B. Gaspar, E. Macintyre, C. Picard, F. D. Bushman, A. Galy, A. Fischer, E. Six, A. J. Thrasher, M. Cavazzana

**Affiliations:** 1 Department of Biotherapy, Hôpital Universitaire Necker-Enfants Malades, Groupe Hospitalier Paris Centre, Assistance Publique-Hôpitaux de Paris, Paris, France; 2 Biotherapy Clinical Investigation Center, Groupe Hospitalier Universitaire Paris Centre, Assistance Publique-Hôpitaux de Paris, INSERM CIC 1416, Paris, France; 3 Clinical Investigation Center CIC 1419, Hôpital Universitaire Necker-Enfants Malades, Groupe Hospitalier Paris Centre, Université de Paris, Assistance Publique-Hôpitaux de Paris, Paris, France; 4 INSERM, UMR_S1176, Université Paris-Saclay, Le Kremlin-Bicêtre, France; 5 Department of Paediatric Immunology, Great Ormond Street Hospital, London, UK; 6 Molecular and Cellular Immunology Section, UCL Great Ormond Street Institute of Child Health, London, UK; 7 Toulouse Institute for Infectious and Inflammatory Diseases (INFINITy), INSERM, CNRS, Toulouse III Paul Sabatier University, Toulouse, France; 8 Ludwig Boltzmann Institute for Rare and Undiagnosed Diseases, Vienna, Austria; 9 Department of Dermatology, Medical University of Vienna, Vienna, Austria; 10 Institute of Immunity and Transplantation, University College London, London, UK; 11 Department of Immunology, Royal Free London Hospitals NHS Foundation Trust, London, UK; 12 Laboratoire d’Hématologie, Assistance Publique-Hôpitaux de Paris, Hôpital Necker-Enfants Malades, Paris, France; 13 EMiLy, INSERM U1160, Institut de Recherche Saint Louis, Université de Paris, Paris, France; 14 Laboratoire d’Immunologie et d’Histocompatibilité, Hôpital Saint-Louis, Assistance Publique-Hôpitaux de Paris, Paris, France; 15 Institut Necker-Enfants Malades (INEM), INSERM U1151, Université Paris Descartes Sorbonne Cité, Paris, France; 16 Laboratory of Onco-Hematology, Assistance Publique-Hôpitaux de Paris, Necker-Enfants Malades University Hospital, Paris, France; 17 Département d’Immunologie, Groupement Hospitalier Pitié-Salpêtrière, Assistance Publique-Hôpitaux de Paris, Paris, France; 18 Centre d’Immunologie et des Maladies Infectieuses-Paris (CIMI-Paris), INSERM, Sorbonne Université, Paris, France; 19 Platelet Immunology Department, INTS, Paris, France; 20 Department of Pediatric Immunology, Hematology and Rheumatology, Assistance Publique-Hôpitaux de Paris, Necker-Enfants Malades University Hospital, Paris, France; 21 Imagine Institute, Université Paris Centre, Paris, France; 22 Human Lymphohematopoiesis Laboratory, Imagine Institute, INSERM UMR 1163, Université de Paris, Paris, France; 23 Service d’Hématologie Adultes, Hôpital Necker-Enfants Malades, Assistance Publique-Hôpitaux de Paris, Centre Université de Paris, Paris, France; 24 Department of Microbiology, University of Pennsylvania School of Medicine, Philadelphia, PA USA; 25 Unité des Technologies Chimiques et Biologiques pour la Santé, CNRS, INSERM, Université de Paris, Paris, France; 26 Clinical Immunology Laboratory, Groupe Hospitalier Universitaire Paris-Sud, Hôpital Kremlin-Bicêtre, Assistance Publique-Hôpitaux de Paris, Le Kremlin-Bicêtre, France; 27 Généthon, Evry, France; 28 Study Center for Primary Immunodeficiencies, Assistance Publique-Hôpitaux de Paris, Necker-Enfants Malades University Hospital, Paris, France; 29 Integrare Research Unit UMRS951, Université Paris-Saclay, Université d’Evry, Inserm, Genethon, Evry, France; 30 Collège de France, Paris, France

**Keywords:** Targeted gene repair, Primary immunodeficiency disorders

Correction to: *Nature Medicine* 10.1038/s41591-021-01641-x, published online 24 January 2022.

In the version of this article initially published, reference 48 (Nurden P. et al. *Br. J. Haematol* **110**, 704–714 (2000)) was incorrect; it should have been Berrou, E. et al. A mutation of the human *EPHB2* gene leads to a major platelet functional defect. *Blood* **132**, 2067–2077 (2018). Further, the Acknowledgements section omitted the statement “We also thank the CIQLE Laënnec-Centre d’Imagerie Quantitative Lyon-Est (France) for expert technical assistance with the electron microscopy studies, and we thank J. C. Bordet (Laboratoire d’Hémostase, Centre de Biologie Est, Hospices Civils de Lyon, Bron, France) for his expertise in transmission electron microscopy and image analysis.” The changes have been made to the HTML and PDF versions of the article.

