# Supplementary material for: Long-term safety and efficacy of lentiviral hematopoietic stem/progenitor cell gene therapy for Wiskott–Aldrich syndrome
Source: Nat Med. 2022 Jan 24;28(1):71–80. doi: 10.1038/s41591-021-01641-x (PMC8799465; doi:10.1038/s41591-021-01641-x)
Supplement: Supplementary file 1 — Supplementary Tables 1–4 and CONSORT flowchart. [file 41591_2021_1641_MOESM1_ESM.pdf]

nature portfolio

https://doi.org/10.1038/s41591-021-01641-x

Supplementary information

Long-term safety and efficacy of lentiviral hematopoietic stem/progenitor cell gene therapy for Wiskott–Aldrich syndrome

In the format provided by the authors and unedited

Supplementary Table 1. Characteristics of the patients before and after GT, and characteristics of the infused gene-corrected autologous cell product

Patient ID	Age at the time of gene therapy	Clinical status before GT	Clinical score before GT	WAS gene mutation	Presence of revertant cells / expression of mutated WASp[a]	Origin of HSCs	Dose of CD34+ cells (x 10^6/kg)	VCN in the transduced CD34+ cells	Follow-up (months)	Clinical status after treatment
**P1**	10 y	Thrombocytopenia, Pjp, molluscum, moderate diffuse eczema, arthropathy, renal disease, recurrent infections	5	401C>T; Ala134Val	-/+	BM	2	0.7	109	A&W, eczema and infections resolved, reduced AI complications, splenectomy 3 y post-GT for ongoing thrombocytopenia , steroids for scleritis
**P2**	15.5 y	Thrombocytopenia, eczema, autoimmunity, severe vasculitis, unable to walk, splenectomized	5	c.1453+1 G>C	+/-	mPB	11	1.3	106	A&W, eczema and infections resolved, improvement of vasculitis, normal physical activity
**P4**	10 mo	Profound refractory thrombocytopenia, eczema	5	c.1295delG, p.G432EfsX13	-/-	BM	7.3	2.8	96	A&W, eczema and infections resolved, one episode of arthritis, bleeding resolved
**P5**	3 y	Profound refractory thrombocytopenia, severe bleeding, eczema	5	c.257G>A, p.R86H	-/-	BM	6.8	0.6	94	A&W, eczema and infections resolved, no major bleeding, on eltrombopag (family decision)
**P6**	7 y	Recurrent epistaxis, eczema, recurrent infections	3	100C>T; Arg34X	+/-	BM	3.1	1.7	89	A&W, eczema and infections resolved, occasional petechiae
**P7**	3.5 y	Severe thrombocytopenia, mild skin vasculitis	5	1271dup(ex10) Leu425Profsx70	-/+	mPB	15	0.6	75	A&W, eczema resolved, frequent bruises, on romiplostim
**P8**	30 y	Cutaneous vasculitis, arthropathy, lymphoproliferation, chronic kidney disease, IS treatment, splenectomized	5	c.931+2T>C	-/-	mPB	3.3	0,8	48	Reduced AI/inflammation and infections, off IS, remained on colchicine; died 4 y post-GT from pneumococcal sepsis and H1N1 influenza
**P9**	11 mo	GI bleeding, severe eczema, severe infections, CMV viremia, failure to thrive	5	c.913 C>T p. (Gln305*) hemozygote c.391G>A p. (Glu131Lys) hemizygote	-/-	mPB	12.3	1,02	52.1	A&W, glomerulonephritis, nephrotic syndrome, eczema resolved, discontinued Ig replacement therapy, on MMF

Abbreviations: WAS, Wiskott-Aldrich syndrome; WASp, Wiskott-Aldrich syndrome protein; y, years; mo, months; HSC, hematopoietic stem cell; VCN, vector copy number; BM, bone marrow; mPB, mobilized peripheral blood; AI, autoimmunity; IS, immunosuppressants; Pjp, *Pneumocystis jiroveci* pneumonia; GI, gastro-intestinal; CMV, cytomegalovirus; A&W, alive and well; Ig, immunoglobulin; MMF, mofetil mycophenolate

[a] Plus or minus signs indicate that revertant cells or mutant protein were either present and detectable (plus) or absent and not detectable (minus)

Supplementary Table 2. Immune response after vaccination.

**Patient ID**	**Vaccine**	**Ig titer**	**Reference values**
P4	Pneumococcus	2.03	>1
	B Hepatitis	410	>10
P5	Measles	276	>16.5
	Poliovirus 1/2	15/30	>5
	Chicken pox	1302	>150
P8	Pneumococcus	Protective responses against 3/13 serotypes	
	Tetanus toxoid	4	>150

Supplementary Table 3. Circulating autoantibodies detected before and after GT.

	Patient 2			Patient 4				Patient 5		Patient 7		
	**Pre-GT**	**4 y**	**6 y**	**Pre-GT**	**3 y**	**5 y**	**6 y**	**Pre-GT**	**6 y**	**Pre-GT**	**2 y**	**4 y**
**Anti-DNA**	neg	neg	neg	neg	neg	neg	neg	neg	neg	neg	neg	neg
**ANA**	1/100 Homogeneous and speckled	neg	1/80 speckled	1/100 speckled	neg	neg	neg	1/100 speckled	neg	1/100 speckled	neg	1/80 speckled
**ANCA**	neg	neg	neg	neg	neg	neg	neg	neg	neg	-	neg	neg
**Anti-RNP (N<230 cpm)**	249 cpm	neg	neg	-	neg	neg	neg	-	neg	-	neg	neg
**Anti-SmB (N<300 cpm)**	153 cpm	neg	neg	-	neg	neg	neg	-	neg	-	neg	neg
**Anti-SSA Ro (N<225 cpm)**	852 cpm	neg	neg	-	neg	neg	neg	-	neg	-	neg	neg
**Anti-SSB (N<180 cpm)**	151 cpm	neg	neg	-	neg	neg	neg	-	neg	-	neg	neg
**Anti-tissue**	-	neg	neg	-	Anti-smooth muscle 1/40*	Anti-smooth muscle 1/40*	neg	-	neg	-	neg	neg

Abbreviations: GT, gene therapy; y, years; neg, negative

*weakly positive, not significant

Supplementary Table 4. Antiplatelet antibodies detected after GT.

**Patient ID**		**Follow-up after GT (months)**	**Date of sampling**	**Platelet count (x10^9/L)**	**Results (MAIPA indirect)**	**Results (Luminex)**
**Circulating antibodies:**						
P2	plasma	84	23/05/2018	47	**GP Ia IIa: positive**<br>**GP IIb IIIa: positive**<br>**GP Ib IX: positive**	negative
	plasma	91	14/11/2018	57	**GP Ia IIa: weakly positive**<br>GP IIb IIIa: negative<br>**GP Ib IX: weakly positive**	negative
P4	serum	60	18/04/2017	18	GP Ia IIa: negative<br>GP IIb IIIa: negative<br>GP Ib IX: negative	negative
	plasma	72	19/04/2018	19	GP Ia IIa: negative<br>GP IIb IIIa: negative<br>GP Ib IX: negative	
	serum	78	29/10/2018	23	GP Ia IIa: negative<br>GP IIb IIIa: negative<br>GP Ib IX: negative	GP Ia IIa: indeterminate
	serum	84	02/05/2019	18	GP Ia IIa: negative<br>GP IIb IIIa: negative<br>GP Ib IX: negative	GP Ia IIa: indeterminate
P5	plasma	55	11/01/2017	11	GP Ia IIa: negative<br>GP IIb IIIa: negative<br>GP Ib IX: negative	negative
P7	plasma	58	12/09/2018	12	GP Ia IIa: negative<br>GP IIb IIIa: negative<br>GP Ib IX: negative	negative
	plasma	79	16/09/2020	12	GP Ia IIa: negative<br>GP IIb IIIa: negative<br>GP Ib IX: **weakly positive**	
P4's mother	serum	-	02/05/2019	238	GP Ia IIa: negative<br>GP IIb IIIa: negative<br>GP Ib IX: negative	
**Platelet-bound antibodies :**						
P2		91	14/11/2018	57	GP Ia IIa: negative<br>**GP IIb IIIa: weakly positive**<br>GP Ib IX: negative	-
P4		78	29/10/2018	23	**GP Ia IIa: positive**<br>**GP IIb IIIa: positive**<br>**GP Ib IX: positive**	**-**
		84	02/05/2019	18	GP Ia IIa: negative<br>GP IIb IIIa: negative<br>GP Ib IX: negative	-
P7		79	16/09/2020		GP Ia IIa: negative<br>GP IIb IIIa: indeterminate<br>GP Ib IX: negative	
P4's mother		-	02/05/2019	238	GP Ia IIa: negative<br>**GP IIb IIIa: weakly positive**<br>GP Ib IX: negative	-

Abbreviations: P, patient; WAS, Wiskott-Aldrich syndrome; GT, gene therapy; GP glycoprotein; MAIPA, Monoclonal Antibody-specific Immobilization of Platelet Antigen technique.

CONSORT

TRANSPARENT REPORTING of TRIALS

CONSORT 2010 Flow Diagram

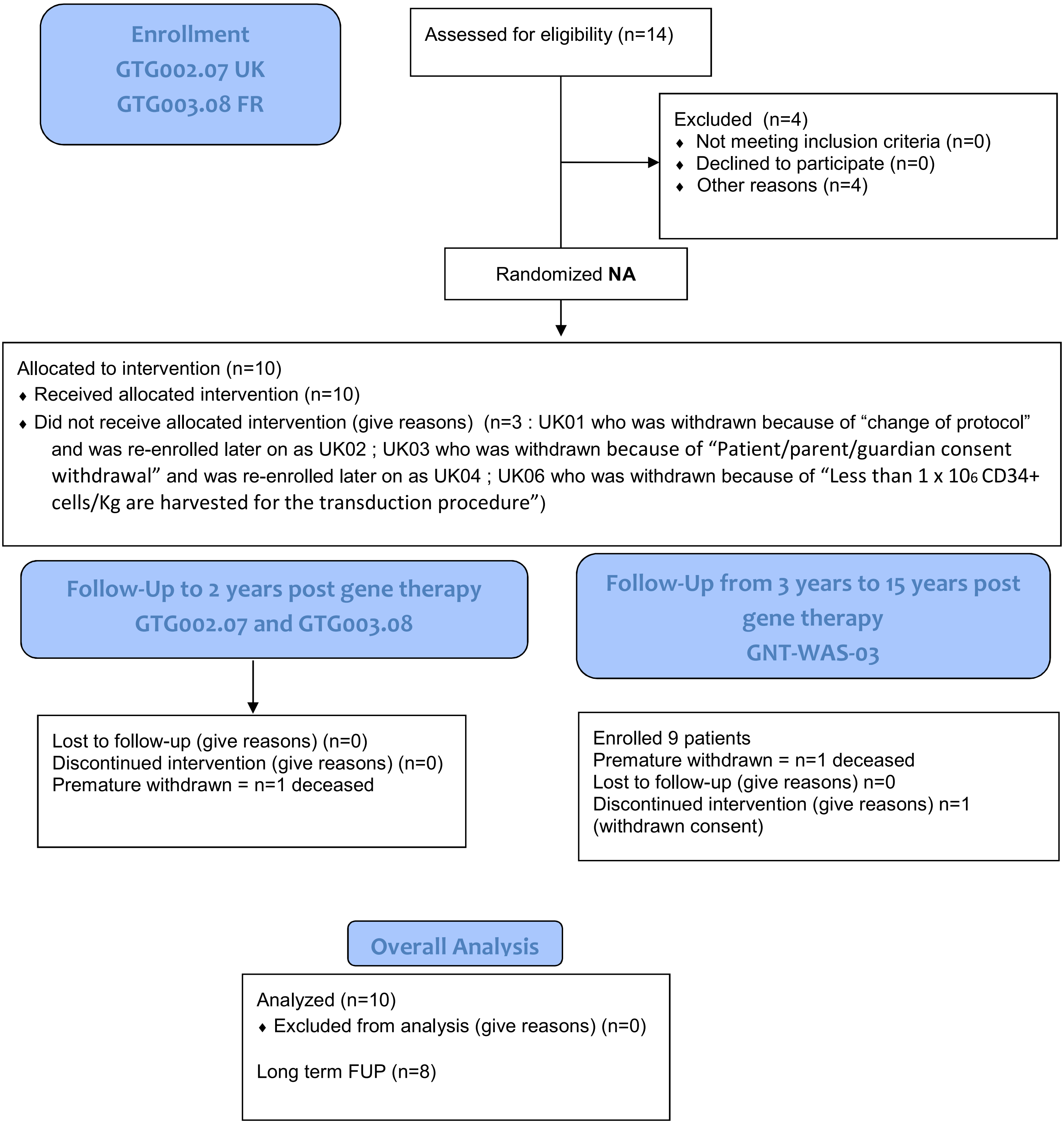

Date of enrollment

GTG002.08 authorized on 2010-02-05, completed on 2017-01-09
GTG002.07 authorized on 2010-01-11, completed on 2019-11-13
GNT-WAS-03 authorized on 2014-05-14, ongoing
