# Supplementary material for: Long-term safety and efficacy of lentiviral hematopoietic stem/progenitor cell gene therapy for Wiskott–Aldrich syndrome
Source: Nat Med. 2022 Jan 24;28(1):71–80. doi: 10.1038/s41591-021-01641-x (PMC8799465; doi:10.1038/s41591-021-01641-x)
Supplement: Supplementary file 2 — Reporting Summary [file 41591_2021_1641_MOESM2_ESM.pdf]

nature research

Corresponding author(s): Pr Marina Cavazzana
Pr Adrian Thrasher

Last updated by author(s): Nov 8, 2021

Reporting Summary

Nature Research wishes to improve the reproducibility of the work that we publish. This form provides structure for consistency and transparency in reporting. For further information on Nature Research policies, see our Editorial Policies and the Editorial Policy Checklist.

Statistics

For all statistical analyses, confirm that the following items are present in the figure legend, table legend, main text, or Methods section.

n/a	Confirmed	
☐	☒	The exact sample size (*n*) for each experimental group/condition, given as a discrete number and unit of measurement
☒	☐	A statement on whether measurements were taken from distinct samples or whether the same sample was measured repeatedly
☐	☒	The statistical test(s) used AND whether they are one- or two-sided<br>*Only common tests should be described solely by name; describe more complex techniques in the Methods section.*
☒	☐	A description of all covariates tested
☒	☐	A description of any assumptions or corrections, such as tests of normality and adjustment for multiple comparisons
☐	☒	A full description of the statistical parameters including central tendency (e.g. means) or other basic estimates (e.g. regression coefficient) AND variation (e.g. standard deviation) or associated estimates of uncertainty (e.g. confidence intervals)
☐	☒	For null hypothesis testing, the test statistic (e.g. *F*, *t*, *r*) with confidence intervals, effect sizes, degrees of freedom and *P* value noted<br>*Give P values as exact values whenever suitable.*
☒	☐	For Bayesian analysis, information on the choice of priors and Markov chain Monte Carlo settings
☒	☐	For hierarchical and complex designs, identification of the appropriate level for tests and full reporting of outcomes
☒	☐	Estimates of effect sizes (e.g. Cohen's *d*, Pearson's *r*), indicating how they were calculated

Our web collection on statistics for biologists contains articles on many of the points above.

Software and code

Policy information about availability of computer code

Data collection	Data were collected manually and transferred to Altizem society for data management through Clintrial software
Data analysis	Figures were prepared with Software R (R core team (2019). R version 4.0.4. R foundation for Statistical Computing, Vienna, Austria. URL https://www.R-project.org/). Shannon diversity index were calculated using the vegan package. Circos plots, showing the various V-gene and J-gene combinations, were designed using the circlize package.<br>Flow cytomety data were analyzed using FlowJo software (TreeStar) (FlowJo v10.5.3). WASp expression was quantified using ImageJ software (ImageJ 1.X). WASP expression western blot was analyzed via Gene Tools software (version 4.03.05.0; Syngene, Cambridge, UK). Statistical analyses were performed using the INSPIIRED pipeline (https://github.com/BushmanLab/INSPIIRED). All the sequence data used in the present study are available in the NCBI Sequence Research Archive (SRA, reference: SRP050221 and PRJNA685802).

For manuscripts utilizing custom algorithms or software that are central to the research but not yet described in published literature, software must be made available to editors and reviewers. We strongly encourage code deposition in a community repository (e.g. GitHub). See the Nature Research guidelines for submitting code & software for further information.

Data

Policy information about availability of data

All manuscripts must include a data availability statement. This statement should provide the following information, where applicable:

- Accession codes, unique identifiers, or web links for publicly available datasets
- A list of figures that have associated raw data
- A description of any restrictions on data availability

Data that support the findings in this study are available from the authors upon agreement of the sponsor (Genethon). Restrictions may apply to the availability of these data before the end of the study as they are part of clinical trials, subject to patient confidentiality and are not public. Integration sites sequence data used in

the present study are available in the NCBI Sequence Research Archive (SRA, reference: SRP050221 and PRJNA685802). TCR NGS sequence data and RCL analyses are available upon request.
Inquiries for access to the study clinical data can be submitted to Dr. S. Abbas, Genethon Clinical Development Department (1bis, Rue de l'Internationale, 91000 EVRY - France, Phone: +33 (0)1 69 47 28 28, Fax: +33 (0)1 69 47 19 46) and inquiries will be adressed within 30 working days.

Field-specific reporting

Please select the one below that is the best fit for your research. If you are not sure, read the appropriate sections before making your selection.

☒ Life sciences ☐ Behavioural & social sciences ☐ Ecological, evolutionary & environmental sciences

For a reference copy of the document with all sections, see nature.com/documents/nr-reporting-summary-flat.pdf

Life sciences study design

All studies must disclose on these points even when the disclosure is negative.

Sample size	This is a long term follow-up study that includes the patients from the two previous studies NCT01347346- and NCT01347242.
Data exclusions	None
Replication	In this clinical study, each sample analyzed is a unique sample.
Randomization	This is a longitudinal follow-up study that includes the patients from two previous non-randomized phase I/II studies NCT01347346- and NCT01347242.
Blinding	Blinding is not applicable for autologous gene therapy.

Reporting for specific materials, systems and methods

We require information from authors about some types of materials, experimental systems and methods used in many studies. Here, indicate whether each material, system or method listed is relevant to your study. If you are not sure if a list item applies to your research, read the appropriate section before selecting a response.

Materials & experimental systems

n/a	Involved in the study	
☐	☒	Antibodies
☐	☒	Eukaryotic cell lines
☒	☐	Palaeontology and archaeology
☒	☐	Animals and other organisms
☐	☒	Human research participants
☐	☒	Clinical data
☒	☐	Dual use research of concern

Methods

n/a	Involved in the study	
☒	☐	ChIP-seq
☒	☐	Flow cytometry
☒	☐	MRI-based neuroimaging

Antibodies

Antibodies used	anti-CD3 monoclonal antibody (mAb) (10 µg/ml OKT3, eBioscience);). Unlabeled a24 mAb (Biolegend) that binds to LFA-1 in its high-affinity conformation at a concentration of 2.5 µg/ml; with phalloidin-AF488 (Invitrogen), and anti-WASp rabbit mAbs (Abcam ab75830). The anti-LFA-1 and WASp Abs were then revealed with anti-mouse AF647-coupled and anti-rabbit AF564-coupled secondary Abs (Invitrogen), respectively. BD Accuri C6 flow cytometer (BD Biosciences). mouse anti-WASp primary antibody (1 µg/mL, clone B-9; Santa Cruz; Heidelberg, Germany) or mouse anti-CD41 (0.2 µg/mL, clone SZ22, used as loading control for normalization; Beckman Coulter). goat anti-mouse IgG Alexa Fluor 555 conjugate (4 µg/mL; Invitrogen); cytoskeletal F-actin with Alexa Fluor 488-phalloidin (0.3 µM; Invitrogen). The patients' antiplatelet antibodies were detected using the "monoclonal antibody-specific immobilization of platelet antigen" technique, according to the manufacturer's instructions (apDia, Turnhout, Belgium).
Validation	Each antibody used had a validated technical data sheet as per manufacturer's website showing positive staining.

Eukaryotic cell lines

Policy information about cell lines

Cell line source(s)	P815 (RRID:CVCL_2154)

Authentication	Cells line was not authenticated
Mycoplasma contamination	Mycoplasma free
Commonly misidentified lines (See ICLAC register)	The cell line used is not listed in the ICLAC database

Human research participants

Policy information about studies involving human research participants

Population characteristics	All the information are detailed in the dedicated clinical trial web page: Clinical trials.gov : NCT01347346, Clinical trials.gov : NCT01347242 ClinicalTrials.gov Identifier: NCT02333760
Recruitment	Participants recruited to the study are WAS patients enrolled and treated in the phase I/II studies conducted in France and United Kingdom (NCT01347346- and NCT01347242) and with signed informed consent.
Ethics oversight	The protocols conducted in France and in the UK were approved by the respective national regulatory authorities : the UK MHRA (Medicines and Healthcare Products Regulatory Agency) with advice from the Gene Therapy Advisory Committee (GTAC) and local R&D commitee ; in France by the ANSM (Agence Nationale de Sécurité du Médicament et des Produits de Santé) with approval from a CPP Committee (Comité pour la Protection des Personnes).

Note that full information on the approval of the study protocol must also be provided in the manuscript.

Clinical data

Policy information about clinical studies

All manuscripts should comply with the ICMJE guidelines for publication of clinical research and a completed CONSORT checklist must be included with all submissions.

Clinical trial registration	Clinical trials.gov : NCT01347346; Clinical trials.gov : NCT01347242; ClinicalTrials.gov : NCT02333760
Study protocol	Data that support the findings is in the study protocol and in the protocol synopsis which is available and is public on https://clinicaltrials.gov/ under NCT number 02333760 , the study protocol is available from the authors upon agreement of the sponsor (Genethon). Restriction may apply to study protocol publication as supplement material due to the confidentiality of data and are not public
Data collection	Data were collected on the study sites (Hospital Necker, Paris, France), GOSH, London and UCLH, London) and monitored by Genethon from Period of 2014 to 2018, and then after “CRA plateforme” ,clinical trial management provider selected by GENETHON , collect data and monitored the trial from 2018 to date . Pharmacovigilance was conducted by AXPharma, Paris, under GENETHON control and validation.
Outcomes	Primary outcomes of the long term study were to establish clinical and biological safety, efficacy and tolerability by evaluating the incidence and type of serious adverse events, the clinical status and biological parameters including lentiviral genomic integration sites in different cells sub-populations from 3 years to 15 years post GT. Secondary outcomes included monitoring the need for additional treatments and T cell repertoire diversity.<br>See Clinical trials.gov : NCT01347346; Clinical trials.gov : NCT01347242; ClinicalTrials.gov : NCT02333760
