# Supplementary material for: Long-term safety and efficacy of lentiviral hematopoietic stem/progenitor cell gene therapy for Wiskott–Aldrich syndrome
Source: Nat Med. 2022 Jan 24;28(1):71–80. doi: 10.1038/s41591-021-01641-x (PMC8799465; doi:10.1038/s41591-021-01641-x)
Supplement: Fig. 5f — Unprocessed western blot. [file 41591_2021_1641_MOESM3_ESM.pdf]

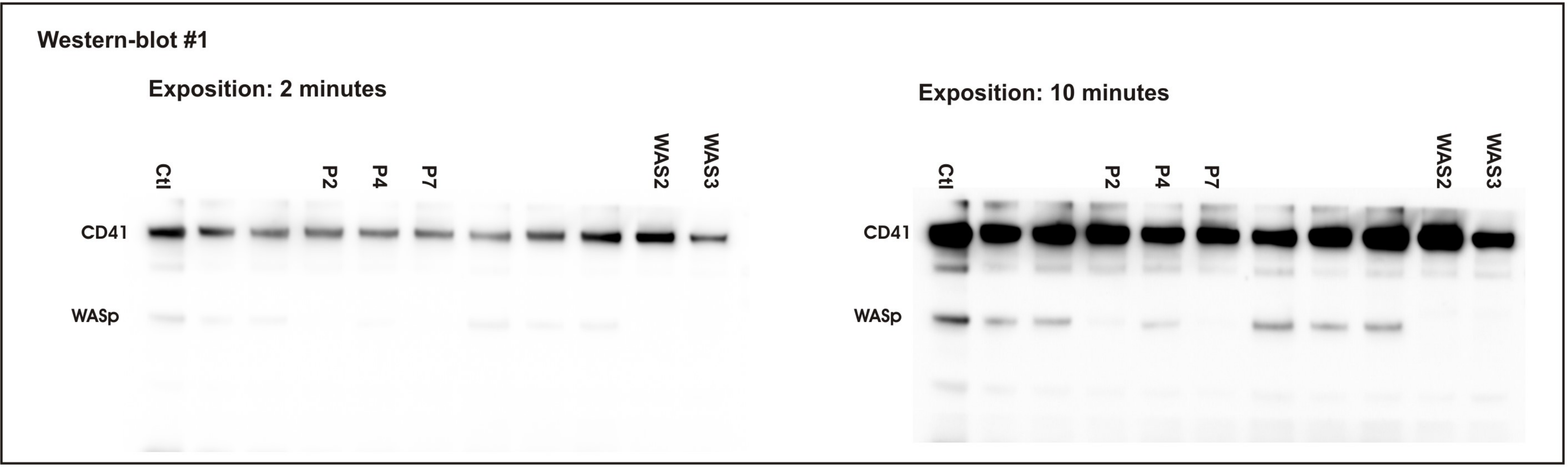
Western-blot #1
Exposition: 2 minutes
Ctl
P2
P4
P7
WAS2
WAS3
CD41
WASp
Exposition: 10 minutes
Ctl
P2
P4
P7
WAS2
WAS3
CD41
WASp

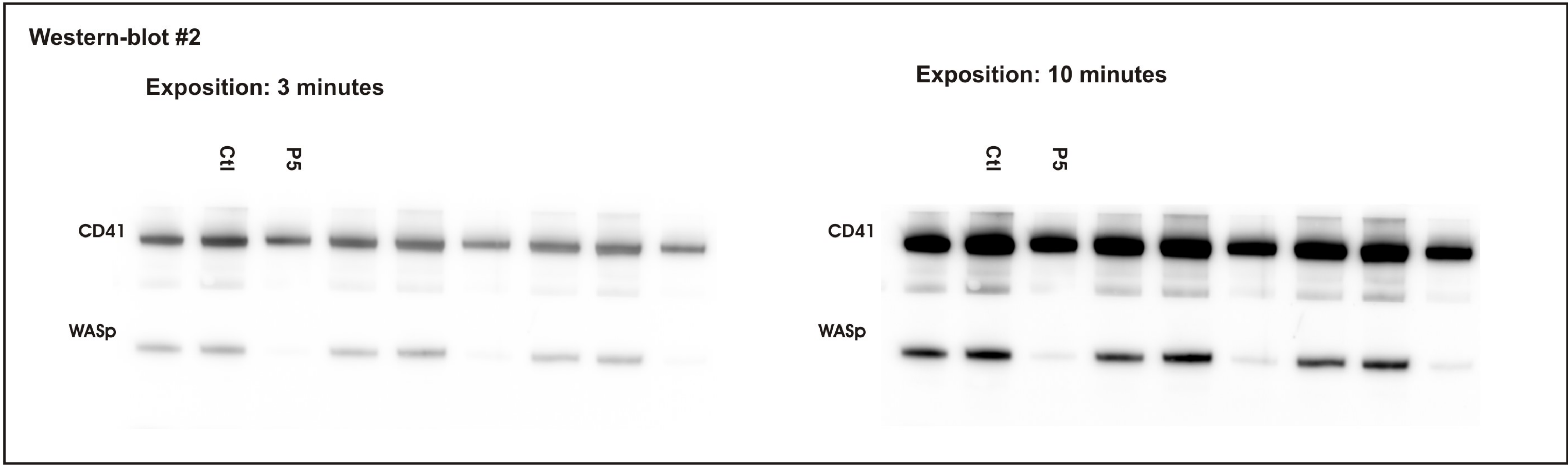
Western-blot #2
Exposition: 3 minutes
Ctl
P5
CD41
WASp
Exposition: 10 minutes
Ctl
P5
CD41
WASp
