# Supplementary figures and images for: Long-term safety and efficacy of lentiviral hematopoietic stem/progenitor cell gene therapy for Wiskott–Aldrich syndrome
Source: Nat Med. 2022 Jan 24;28(1):71–80. doi: 10.1038/s41591-021-01641-x (PMC8799465; doi:10.1038/s41591-021-01641-x)

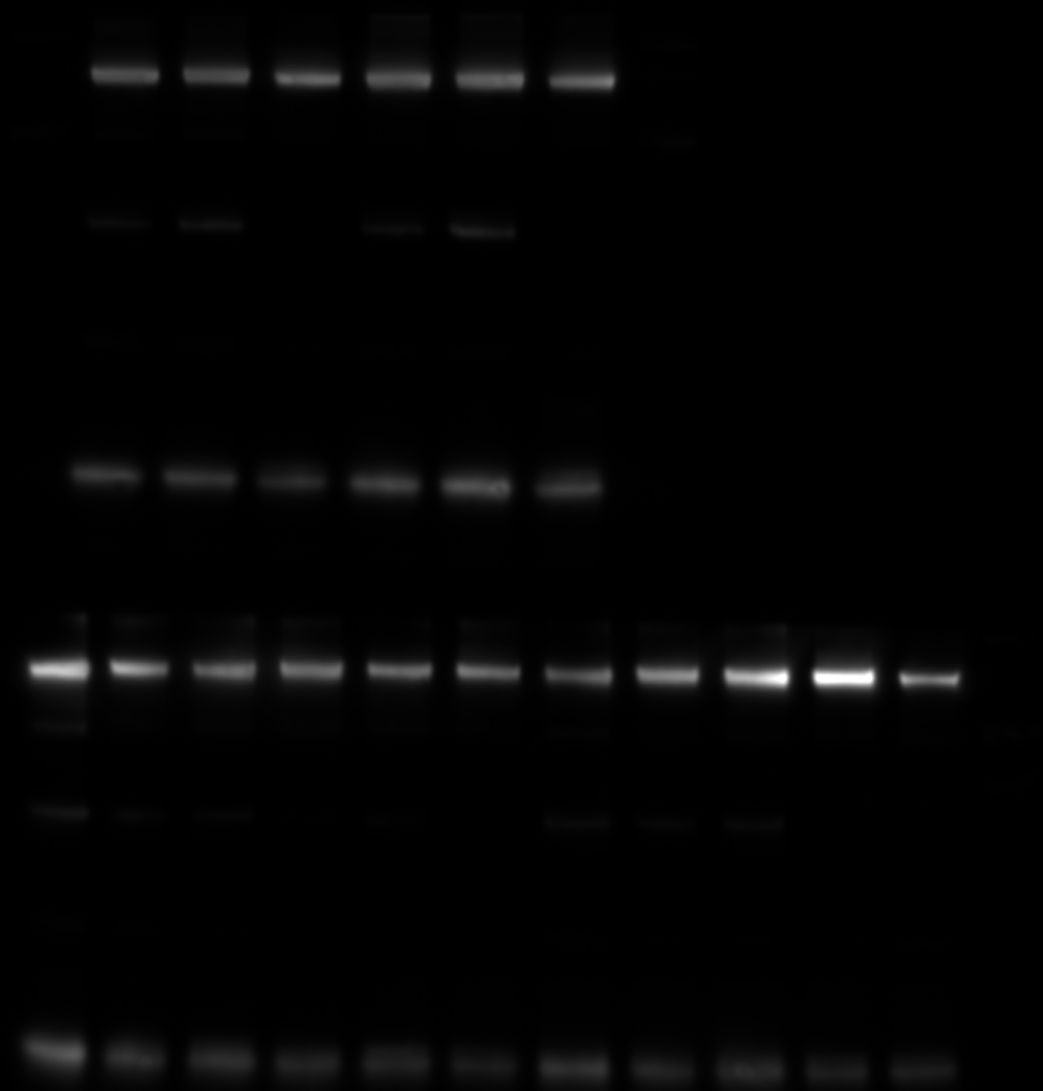

Supplement: Fig. 5f — Unprocessed western blot. [file 41591_2021_1641_MOESM4_ESM.pdf]

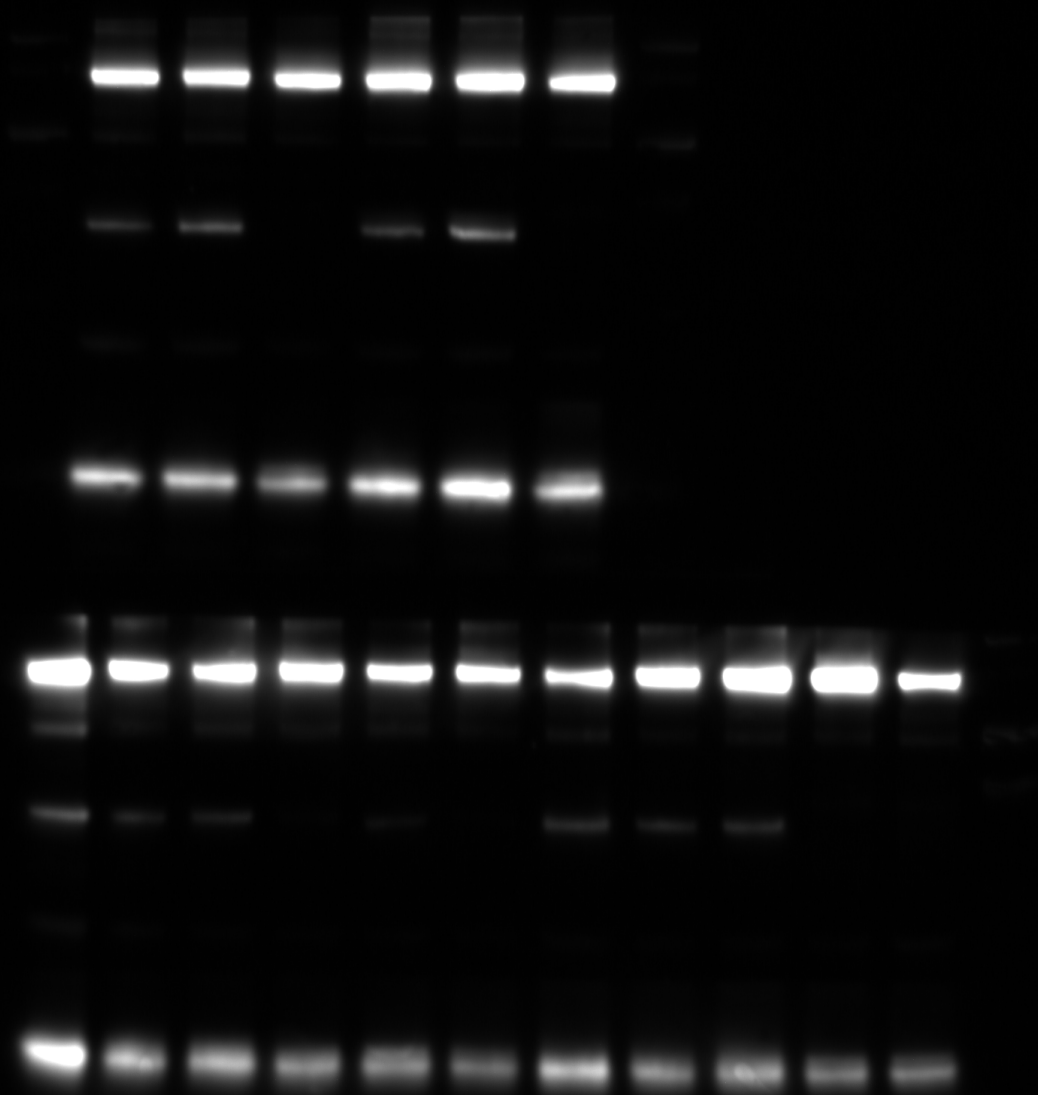

Supplement: Fig. 5f — Unprocessed western blot. [file 41591_2021_1641_MOESM5_ESM.pdf]

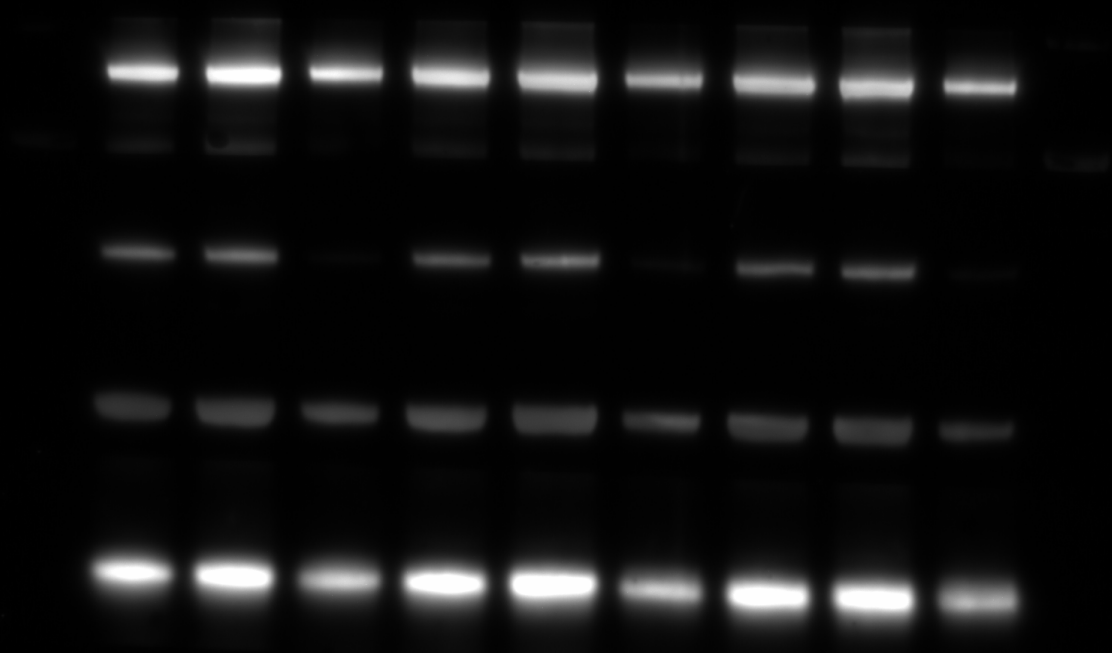

Supplement: Fig. 5f — Unprocessed western blot. [file 41591_2021_1641_MOESM6_ESM.pdf]

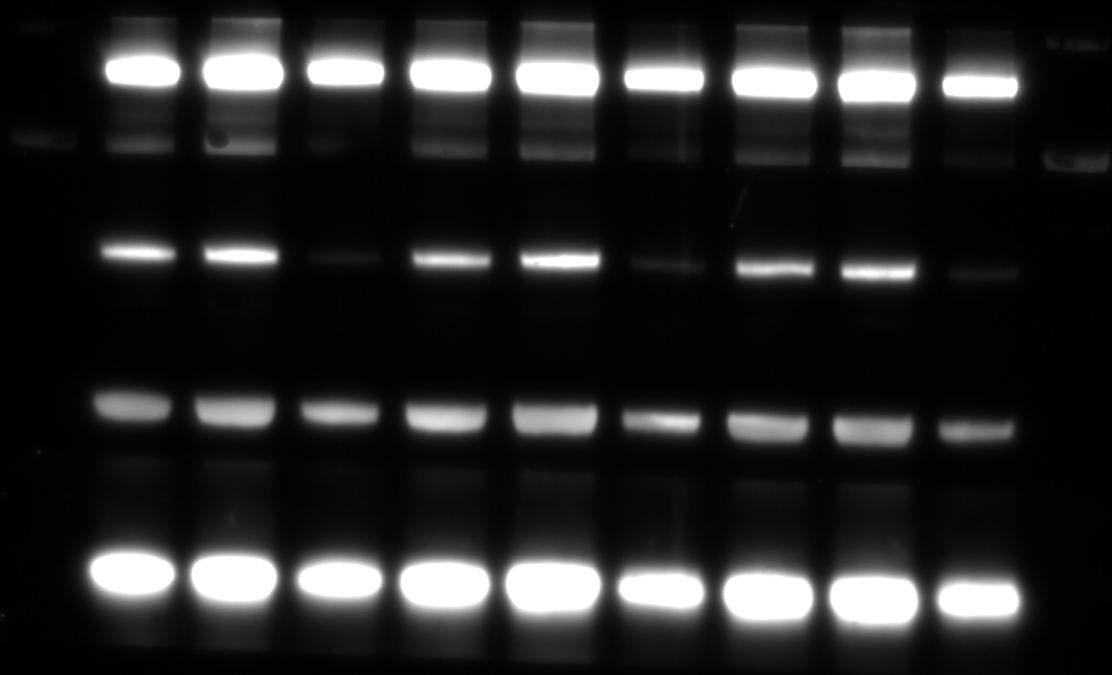

Supplement: Fig. 5f — Unprocessed western blot. [file 41591_2021_1641_MOESM7_ESM.pdf]
